# MUTYH Actively Contributes to Microglial Activation and Impaired Neurogenesis in the Pathogenesis of Alzheimer's Disease

**DOI:** 10.1155/2021/8635088

**Published:** 2021-12-21

**Authors:** Yuri Mizuno, Nona Abolhassani, Guianfranco Mazzei, Kunihiko Sakumi, Takashi Saito, Takaomi C. Saido, Toshiharu Ninomiya, Toru Iwaki, Ryo Yamasaki, Jun-ichi Kira, Yusaku Nakabeppu

**Affiliations:** ^1^Division of Neurofunctional Genomics, Department of Immunobiology and Neuroscience, Medical Institute of Bioregulation, Kyushu University, Fukuoka 812-8582, Japan; ^2^Department of Neurology, Neurological Institute, Graduate School of Medical Sciences, Kyushu University, Fukuoka 812-8582, Japan; ^3^Department of Neurocognitive Science, Institute of Brain Science, Nagoya City University Graduate School of Medical Science, Aichi 467-8601, Japan; ^4^Laboratory for Proteolytic Neuroscience, RIKEN Center for Brain Science, Saitama 351-0198, Japan; ^5^Department of Epidemiology and Public Health, Graduate School of Medical Sciences, Kyushu University, Fukuoka 812-8582, Japan; ^6^Department of Neuropathology, Graduate School of Medical Sciences, Kyushu University, Fukuoka 812-8582, Japan; ^7^Translational Neuroscience Center, Graduate School of Medicine, and School of Pharmacy at Fukuoka, International University of Health and Welfare, Fukuoka 831-8501, Japan

## Abstract

Oxidative stress is a major risk factor for Alzheimer's disease (AD), which is characterized by brain atrophy, amyloid plaques, neurofibrillary tangles, and loss of neurons. 8-Oxoguanine, a major oxidatively generated nucleobase highly accumulated in the AD brain, is known to cause neurodegeneration. In mammalian cells, several enzymes play essential roles in minimizing the 8-oxoguanine accumulation in DNA. MUTYH with adenine DNA glycosylase activity excises adenine inserted opposite 8-oxoguanine in DNA. MUTYH is reported to actively contribute to the neurodegenerative process in Parkinson and Huntington diseases and some mouse models of neurodegenerative diseases by accelerating neuronal dysfunction and microgliosis under oxidative conditions; however, whether or not MUTYH is involved in AD pathogenesis remains unclear. In the present study, we examined the contribution of MUTYH to the AD pathogenesis. Using postmortem human brains, we showed that various types of *MUTYH* transcripts and proteins are expressed in most hippocampal neurons and glia in both non-AD and AD brains. We further introduced MUTYH deficiency into *App*^NL-G-F/NL-G-F^ knock-in AD model mice, which produce humanized toxic amyloid-*β* without the overexpression of APP protein, and investigated the effects of MUTYH deficiency on the behavior, pathology, gene expression, and neurogenesis. MUTYH deficiency improved memory impairment in *App*^NL-G-F/NL-G-F^ mice, accompanied by reduced microgliosis. Gene expression profiling strongly suggested that MUTYH is involved in the microglial response pathways under AD pathology and contributes to the phagocytic activity of disease-associated microglia. We also found that MUTYH deficiency ameliorates impaired neurogenesis in the hippocampus, thus improving memory impairment. In conclusion, we propose that MUTYH, which is expressed in the hippocampus of AD patients as well as non-AD subjects, actively contributes to memory impairment by inducing microgliosis with poor neurogenesis in the preclinical AD phase and that MUTYH is a novel therapeutic target for AD, as its deficiency is highly beneficial for ameliorating AD pathogenesis.

## 1. Introduction

Alzheimer's disease (AD) is pathologically characterized by brain atrophy, amyloid plaques, neurofibrillary tangles (NFTs), and loss of neurons, thereby causing a progressive decline in the cognitive function [1]. However, neither precise pathogenesis nor fundamental treatment for AD has been established yet. Accumulating evidence indicates that aging and gender are major risk factors for AD [2, 3], and it has been established that those risk factors are tightly associated with increased oxidative stress in AD brains [4].

The accumulation of various oxidized molecules of lipids, proteins, and nucleic acids is increased in the AD brain [4–8]. Among those, 8-oxo-7,8-dihydroguanine (8-oxoguanine, 8-oxoG), an oxidized form of guanine, a major oxidation product in DNA, highly accumulates in AD brains and is recognized as the most pronounced marker of oxidative stress [4, 8–12].

8-OxoG accumulated in cellular DNA can pair with adenine as well as cytosine during replication or transcription, thus causing mutagenesis or cell death [13–16]. In mammalian cells, there are three enzymes that play major roles in minimizing the accumulation of 8-oxoG in DNA. MTH1, also known as NUDT1, with 8-oxo-2′-deoxyguanosine triphosphatase (8-oxo-dGTPase) efficiently hydrolyzes the 8-oxo-dGTP generated in the nucleotide pool to 8-oxo-dGMP and pyrophosphate, thereby preventing the incorporation of 8-oxoG into DNA [17]; OGG1 with 8-oxoG DNA glycosylase excises 8-oxoG opposite cytosine in DNA, thereby preventing the accumulation of 8-oxoG in DNA [18]; and MUTYH, with its adenine DNA glycosylase activity, excises adenine inserted opposite 8-oxoG in DNA, thus initiating base excision repair [19].

The expression of the human mitochondrial form of OGG1 is reported to be significantly reduced and associated with NFTs and dystrophic neurites in the AD brain [20], and serum levels of OGG1 are decreased in AD patients [21]. In AD brains, synaptic levels of MTH1 are significantly reduced in the CA1 and CA3 fields [22, 23]. Indeed, MTH1/OGG1-double deficiency in 3xTg-AD model mice significantly increased the 8-oxoG accumulation in hippocampal and cortical neurons, accompanied by exacerbated AD pathology [24]. Under conditions of increased oxidative stress, reduced OGG1 and/or MTH1 levels in the AD brain may largely account for the increased accumulation of 8-oxoG in the AD brain. However, there have been no reports examining the MUTYH expression in the AD brain or MUTYH deficiency in AD model mice; as such, the involvement of MUTYH in AD pathogenesis remains unclear.

Since we previously showed that MUTYH deficiency improves neurodegeneration in several mouse models, including 3-nitropropionic acid-induced striatum degeneration [25] and inherited retinal degeneration [26], MUTYH likely does not protect the brain, instead playing a deleterious role during the neurodegenerative process [14, 27].

In the present study, we explored whether or not MUTYH contributes to the pathogenesis of AD by examining the MUTYH expression in postmortem human brains and investigating the effects of MUTYH deficiency in *App*^NL-G-F/NL-G-F^ knock-in AD model mice [28].

## 2. Materials and Methods

### 2.1. Postmortem Human Brain Samples

For the immunohistochemical analysis, postmortem brain tissues were obtained from three female AD and three female control (non-AD) autopsy samples of Hisayama residents in 2014, and formalin-fixed brain samples were embedded in paraffin. The age range was 72-92 years old (Supplementary Table S1). The assessment of AD pathology was conducted according to the Consortium to Establish a Registry for Alzheimer's Disease (CERAD) guidelines [29] and the Braak stage [30].

The use of human postmortem brain tissue was approved by the Ethics Committee of the Faculty of Medicine, Kyushu University, Fukuoka, Japan, and was performed in accordance with the ethical standards described in the latest revision of the Declaration of Helsinki. Written informed consent for all subjects was obtained from their families.

### 2.2. Immunohistochemical Analyses of Postmortem Human Brain Samples

For the immunohistochemical detection of MUTYH, formalin-fixed postmortem brain samples (three female AD and three female non-AD patients) embedded in paraffin were cut at a thickness of 6 *μ*m. The sections were deparaffinized and rehydrated and then pretreated for antigen retrieval by autoclaving in 0.01 mol/L citrate buffer (pH 6.0) at 121°C for 10 min. Subsequently, sections were incubated with the rabbit anti-MUTYH antibody (3.6 *μ*g/ml), which had previously been raised against recombinant human MUTYH protein in our laboratory [19], in 5% normal goat serum-containing phosphate-buffered saline (PBS; 137 mM NaCl, 2.68 mM KCl, 8.1 mM Na_2_HPO_4_, 1.47 mM KH_2_PO_4_, pH 7.6) overnight at 4°C.

For the immunohistochemical detection of 8-oxoG, the brain samples were cut at a thickness of 4 *μ*m. To detect 8-oxoG in mitochondrial DNA, the sections were pretreated with RNase (5 mg/ml; R4642; Sigma-Aldrich, Tokyo, Japan) for 60 min at 37°C and then with proteinase K (1.2 *μ*g/ml; 162-22751; FUJIFILM Wako Pure Chemical Corporation, Osaka, Japan) for 15 min at room temperature for antigen retrieval. To detect 8-oxoG in nuclear DNA, the retrieved sections were further pretreated with 4 N HCl for 20 min at room temperature to denature the nuclear DNA. The sections were then incubated with mouse anti-8-oxo-dG (1 : 100, NS45.1; Japan Institute for the Control of Aging, Nikken Seil Co., Ltd., Shizuoka, Japan) overnight at 4°C. To prepare the mouse anti-8-oxo-dG preadsorbed by antigen, the antibody was mixed with 8-oxo-dG (Sigma-Aldrich) or 2′-deoxyguanosine (dG) (7132; Yamasa Corporation, Chiba, Japan) at a molar ratio of 1 : 100 and then was incubated for 12 h at 4°C.

Rinsed sections were treated with 0.3% H_2_O_2_ in methanol for 30 min at room temperature and then processed by the Universal Immuno-peroxidase Polymer method using N-Histofine Simple Stain MAX PO® (Anti-rabbit, 414141F; Nichirei Biosciences Inc., Tokyo, Japan) or EnVision+ System-HRP-labeled polymer (Anti-mouse, K4001; Agilent, Santa Clara, CA, USA). The bound peroxidase was detected using the 3,3′-diaminobenzidine (DAB; Dojindo, Kumamoto, Japan) reaction, and sections were lightly counterstained with hematoxylin. Digital images were acquired using an AxioImager A1 microscope, equipped with an AxioCam charge-coupled device camera, and the AxioVision 4.9 imaging software program (Carl Zeiss Microscopy, Tokyo, Japan) as well as a Nikon Eclipse 80i microscope with a virtual slice module in the Stereo Investigator software program (MBF Bioscience, Williston, VT, USA). Immunoreactivity and the number of cells were quantified using the Fiji software program (NIH; http://fiji.sc/).

### 2.3. Western Blotting of Postmortem Human Brain Samples

Hippocampal extracts (13.5 *μ*g total protein/lane) previously prepared from postmortem brains (four female AD and four female non-AD patients) [31] were subjected to 10% sodium dodecyl sulfate (SDS) polyacrylamide gel electrophoresis and transferred onto an Immobilon-P PVDF membrane (IPVH00010; Merck, Darmstadt, Germany). The membrane was incubated for 1 h at room temperature in Tris-buffered saline (TBS; 10 mM Tris-HCl pH 7.5, 0.9% NaCl) containing 0.1% Tween-20 (TBST) and 5% nonfat dried milk (Megmilk Snow Brand, Tokyo, Japan). The membrane was then incubated in TBST with 1% nonfat dried milk containing a rabbit anti-MUTYH antibody [19] (2 *μ*g/ml) overnight at 4°C with gentle shaking. Subsequently, the membrane was washed and incubated in TBST with 1% nonfat dried milk containing an anti-rabbit IgG HRP-linked goat antibody (1 : 3000, 7074S; Cell Signaling Technology, Inc., Danvers, MA, USA) for 1 h at room temperature.

Next, bound HRP-linked antibodies on the blots were detected by the chemiluminescence method with EzWestLumi plus (WSE-7120; ATTO, Tokyo, Japan). Digitized images were obtained with an AE-9300 Ez-CaptureMG (ATTO) and analyzed using the densitograph software program CS Analyzer 3 (ATTO). To obtain a loading control, the membrane was then stripped and reprobed with mouse anti-*β*-actin (1 : 5000, A5316-2ML; Sigma-Aldrich) and HRP-linked protein A (1 : 10000, NA9120-1ML; Cytiva, Tokyo, Japan).

### 2.4. The Analysis of *MUTYH* Transcripts in RNA Sequencing Data Obtained from Postmortem Human Brain Samples

We performed Illumina high-throughput RNA sequencing using hippocampal RNA samples that had been prepared previously from 10 non-AD and 8 AD patients [31], and the obtained data (accession number GSE173955, Abolhassani et al., in preparation) were analyzed using the StringTie (v2.1.4) software program to obtain the expression profiles for both the *MUTYH* gene and its transcript variants along with their transcripts per million (TPM) values. The expression difference analysis was performed by the likelihood ratio test using the R package edgeR. The IGV software program was used to visually explore the data and their sequences.

### 2.5. Animals

Previously established heterozygous *App*^+/NL-G-F^ knock-in mice carrying a humanized amyloid-*β* (A*β*) sequence (G676R, F681Y, and R684H) with three pathogenic mutations—Swedish (KM670/671NL), Beyreuther/Iberian (I716F), and Arctic (E693G) [28]—were backcrossed with C57BL/6J for 10 generations to obtain *App*^NL-G-F/NL-G-F^ mice, which were maintained by inbred crossing. Previously established *Mutyh*^+/-^ mice [32, 33] were backcrossed with C57BL/6J for over 39 generations. *App*^+/NL-G-F^·*Mutyh*^+/-^ mice obtained by crossing *App*^NL-G-F/NL-G-F^ and *Mutyh*^+/-^ mice were mated to obtain wild-type, *App*^NL-G-F/NL-G-F^, and *App*^NL-G-F/NL-G-F^·*Mutyh*^−/−^ mice. These three strains of mice were maintained as inbred strains.

The primer sequences used to determine the genotype were as follows: *Mutyh*^+^ allele (172 bp): 5′-GACCTGGTTCGGTCTCTTCC-3′ and 5′-GCAGTAGACACAGCTGCAT-3′, and *Mutyh*^−^ allele (549 bp): 5′-AGTTGTTGACGCTAGGGCTC-3′ and 5′-GCAGTAGACACAGCTGCAT-3′.

The mice were maintained in an air-conditioned specific pathogen-free room at 22°C with a 12 h light-dark cycle (lights on 8:00 am, off at 8:00 pm) and given free access to food and water. All animal experimental procedures were reviewed and approved by the Animal Care and Use Committee at Kyushu University (approval numbers A20-080-0, A-30-077-0, A19-051-0, A29-124-0, A29-121-0, A28-013-0, and A27-257-0) and carried out in accordance with the relevant guidelines and regulations. All animal experimental protocols were performed with adherence to the Animal Research: Reporting of In Vivo Experiments (ARRIVE) guidelines (https://www.nc3rs.org.uk/arrive-guidelines).

### 2.6. Behavioral Analyses

**Figure 1:**
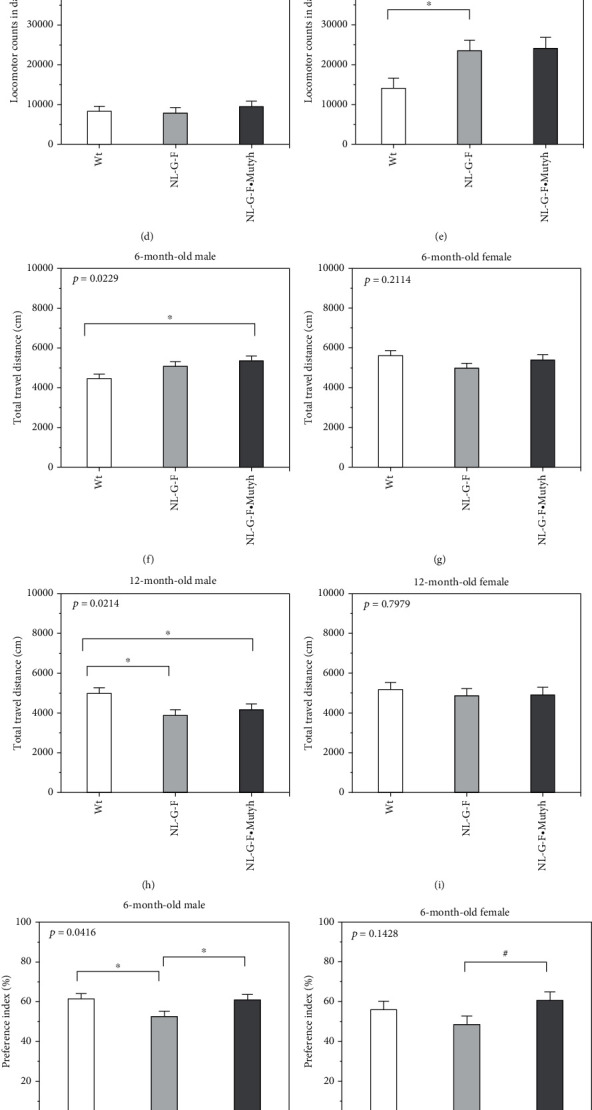
A behavioral analysis of wild-type, *App*^NL-G-F/NL-G-F^, and *App*^NL-G-F/NL-G-F^·*Mutyh*^−/−^ mice. (a) Timelines for the behavioral analyses. After being individually housed for at least seven days, mice were randomly assigned to group A or group B for the behavioral analyses. HC = spontaneous locomotor activity in the home cage; OF = open-field test; NOR = novel object recognition test. (b–e) Spontaneous locomotor activity. Locomotor counts in the dark phase averaged over three days (8:00 pm to 8:00 am) are shown. Wild-type (Wt), *App*^NL-G-F/NL-G-F^ (NL-G-F), and *App*^NL-G-F/NL-G-F^·*Mutyh*^−/−^ (NL-G-F·Mutyh). (f–i) Open-field test. The total travel distance (cm) during 5 min is shown. (j–m) Novel object recognition test. The preference index is shown. (b, f, j) Six-month-old male mice. (c, g, k) Six-month-old female mice. (d, h, l) Twelve-month-old male mice. (e, i, m) Twelve-month-old female mice. The data are expressed as the mean ± SEM. *n* = 13-15 per group. Statistical analyses were performed with a one-way ANOVA (the *p* value is shown in each bar graph) followed by post hoc Student's *t*-test. ^∗^*p* < 0.05. In six-month-old female mice, a one-way ANOVA revealed marginal significance in spontaneous locomotor activity (c) and the novel object recognition test (k), so Hsu's MCB was performed. ^#^*p* < 0.05.

An open-field test and novel object recognition test were performed from 9:00 to 20:00, while spontaneous locomotor activity was measured continuously for three days. At least 13 mice were randomly collected for each age, sex, and genotype, and mice with cancer or skin disease and mice that died during the behavioral analyses were excluded. Mice were individually housed for at least one week before the behavioral test and then assigned randomly to either group A, which started with spontaneous locomotor activity measurement, or group B, which started with the open-field test (Figure 1(a)).

The experiment data were analyzed in a blinded manner. Detailed procedures can be found online in Supplementary Materials.

### 2.7. Microarray Analyses of Mouse Brain Samples

Microarray analyses were performed using hippocampus RNA prepared from six-month-old *App*^NL-G-F/NL-G-F^ and *App*^NL-G-F/NL-G-F^·*Mutyh*^−/−^ mice together with the corresponding wild-type mice, respectively (three females per group), using the GeneChip™ Mouse Gene 2.0 ST Array (Thermo Fisher Scientific, Waltham, MA, USA), as previously described [34]. Generated CEL files were input into Affymetrix's Transcriptome Analysis Console (TAC4.0), and a gene level differential expression analysis was performed according to the user's guide. Gene-level estimates of microarray data were subjected to one-way analysis of variance (ANOVA) with eBayes correction among wild-type, *App*^NL-G-F/NL-G-F^ and *App*^NL-G-F/NL-G-F^·*Mutyh*^−/−^ samples, and 1164 transcript clusters were found to be significantly altered (raw expression intensity > 50, *F*-test, *p* value < 0.05). The lists of transcript clusters (138) significantly altered between wild-type vs. *App*^NL-G-F/NL-G-F^ and *App*^NL-G-F/NL-G-F^ vs. *App*^NL-G-F/NL-G-F^·*Mutyh*^−/−^ samples (*p* < 0.05) were further analyzed using the Database for Annotation, Visualization and Integrated Discovery ver. 6.8 (DAVID). All microarray data were deposited in the GEO database (accession numbers GSE157161 and GSE157766).

### 2.8. Real-Time Quantitative Reverse Transcription Polymerase Chain Reaction (qRT-PCR) of Mouse Hippocampus RNA Samples

RNA samples were reverse-transcribed to first-strand cDNA, as previously described [34]. qRT-PCR was performed to measure the *Cd68* mRNA levels using a Thermal Cycler Dice® Real-Time System Single (Takara Bio Inc.) with 5 ng cDNA and 200 nM of primers for *Cd68* (forward: ACACTTCGGGCCATGTTTCT and reverse: GGGGCTGGTAGGTTGATTGT) and Thunderbird® SYBR® qPCR Mix (QPS-201, Toyobo, Osaka, Japan) in triplicate. The relative expression of *Cd68* was obtained using the second derivative maximum (SDM) standard curve cycle threshold (ct), and the ΔΔCT method was used to calculate values. *Gapdh* was used as an internal control [34].

### 2.9. Immunoblot Analyses of the Mouse Brain Samples

Denatured protein samples (60 *μ*g total protein/lane) prepared from mouse brains as described in Supplementary Materials and Methods were subjected to 8% SDS polyacrylamide gel electrophoresis and transferred onto a 0.2 *μ*m nitrocellulose membrane (10600001; Cytiva). The membrane was incubated in TBST containing 5% nonfat dried milk and then in TBST with 1% nonfat dried milk containing a rabbit anti-MUTYH antibody (1 : 1000, 19650-1-AP; Proteintech Group, Inc., Rosemont, IL, USA) overnight at 4°C. The membrane was then incubated further in TBST with 1% nonfat dried milk containing an anti-rabbit IgG HRP-linked goat antibody and processed as described in Section 2.3.

For dot blot analyses of insoluble A*β* in mouse brain samples, SDS-insoluble A*β* was extracted by formic acid (FA) as described previously [35–38]. In brief, frozen brain samples from four six-month-old female mice for each group were sonicated in 2× SDS sample buffer (130 mM Tris-HCl pH 6.8, 4% SDS, 10% glycerol, 4% 2-mercaptoethanol, and 0.01% bromophenol blue) and centrifuged at 100,000 g for 30 min at 20°C (Optima TLX Ultracentrifuge, TLA55 rotor; Beckman Coulter, Inc., Brea, CA, USA). The remaining pellet was washed once and then extracted by sonication in 70% FA and centrifuged at 100,000 g for 30 min at 20°C. The supernatant was freeze-dried (SC100 Speedvac concentrator, RT100A refrigerated condensation trap; Thermo Fisher Scientific), dissolved in dimethyl sulfoxide (047-29353; FUJIFILM Wako Pure Chemical Corporation), and stored frozen at -80°C until use.

To prepare dot blots, a prewet 0.2 *μ*m nitrocellulose membrane was placed in a Bio-Dot Microfiltration Apparatus (1706545; Bio-Rad Laboratories, Inc., Hercules, CA, USA). After rehydrating the membrane using TBS, appropriate wells were filled with the FA-extracted fraction (0.5 *μ*l sample diluted in 50 *μ*l of TBS). After the entire sample had been filtered through the membrane by gravity flow, the membrane was removed from the apparatus. The membrane was then boiled in PBS at 95°C for 5 min and incubated for 1 h at room temperature, first in TBST containing 5% nonfat dried milk and then in TBST with 1% nonfat dried milk containing mouse anti-*β*-amyloid, 1-16 antibody (1 : 1000, 803002; BioLegend, Inc., San Diego, CA, USA) overnight at 4°C with gentle shaking. The membrane was then incubated further in TBST with 1% nonfat dried milk containing an anti-mouse IgG HRP-linked goat antibody (1 : 4000, 7076; Cell Signaling Technology, Inc.) for 1 h at room temperature and processed as described in Section 2.3.

### 2.10. Immunohistochemical Analyses of Mouse Brain Tissues

Mice were anesthetized with a combination of medetomidine (0.3 mg/kg), midazolam (4.0 mg/kg), and butorphanol (5.0 mg/kg) and then perfused with saline and 4% paraformaldehyde in PBS. The brain was removed, and either frozen brain blocks were prepared as previously described [39] and stored at -80°C until use, or the brain was embedded in paraffin. Frozen brain blocks were cut on a cryostat to a thickness of 40 *μ*m and collected as free-floating sections. To evaluate microgliosis, free-floating sections were blocked in 1× Block Ace solution (UKB80; Dainippon Pharmaceutical, Osaka, Japan) for 30 min at room temperature and then incubated with rabbit anti-CD68 (1 : 1000, ab125212; Abcam plc, Cambridge, UK) overnight at 4°C with gentle shaking. To evaluate A*β* plaques, free-floating sections were also blocked in 2× Block Ace solution for 2 h at room temperature and then incubated with mouse anti-human A*β* 82E1 (1 : 2000, 10323; IBL, Gumma, Japan) overnight at 4°C. Free-floating sections were pretreated with RNase (5 mg/ml) for 60 min at 37°C to detect 8-oxoG in mitochondrial DNA and additionally pretreated with 2 N HCl for 60 min at room temperature to detect 8-oxoG in nuclear DNA. The sections were then blocked in 2× Block Ace solution for 1 h at room temperature and incubated with mouse anti-8-oxo-dG (1 : 100) overnight at 4°C.

For the immunohistochemical detection of MUTYH protein, mouse brains embedded in paraffin were cut to a thickness of 4 *μ*m. The sections were boiled in HistoVT one (06380-05; Nacalai Tesque, Inc., Kyoto, Japan) for 20 min at 90°C for antigen retrieval and then blocked in 1× Block Ace solution for 1 h at room temperature and incubated with the rabbit anti-MUTYH antibody (1 : 500, ab228722; Abcam plc) overnight at 4°C. The sections were subsequently processed using a Vector ABC kit (PK-6100; Vector Laboratories, Burlingame, CA, USA) with a proper biotinylated secondary antibody. The DAB (SK-4100; Vector Laboratories) reaction was then used to visualize the bound secondary antibody.

Views of the entire coronal section were obtained using a Nikon Eclipse 80i microscope with a virtual slice module in the Stereo Investigator software program (MBF Bioscience). Immunoreactivity was quantified using Fiji (NIH, http://fiji.sc). The minimal signal intensity was subtracted from the mean signal intensity of 8-oxo-dG immunoreactivity in the region of interest to obtain the 8-oxoG index.

### 2.11. 5-Bromo-2′-deoxyuridine (BrdU) Labeling and Stereology

To identify proliferating cells in the brain, BrdU (50 mg/kg, B5002; Sigma-Aldrich) dissolved in 0.9% NaCl solution was intraperitoneally injected twice a day for 3 consecutive days. One day after the last injection, brain samples were prepared as described above and stored at -80°C until used. Brain blocks were cut at a thickness of 40 *μ*m as free-floating sections. Sections were incubated in 50% formamide in 2× SSC (300 mM NaCl, 30 mM sodium citrate, pH 7.0) for 2 h at 65°C and treated with 2 N HCl at 37°C for 30 min to denature nuclear DNA, followed by treatment with Tris-HCl (pH 7.5) for 10 min. After being blocked in Block Ace, sections were incubated with mouse anti-BrdU (1 : 800, BMC9318; Roche Diagnostics K.K, Tokyo, Japan) overnight at 4°C. The next day, sections were processed using the Vector ABC kit with an appropriate biotinylated secondary antibody.

To estimate the total number of BrdU-positive cells in the subgranular zone (SGZ) and granule cell layer (GCL) in the hippocampus, we used a modified version of the fractionator principle previously described [40]. In brief, a total of 11 serial sections along the septo-temporal axis of the hippocampus, 200 *μ*m apart (bregma -1.35 to -3.51 mm; every 5 sections), were processed using a semiautomatic stereology system (Stereo Investigator; MBF Bioscience). All BrdU-positive cells in the whole area of the SGZ and GCL in the sections, with exclusion of BrdU-labeled cells present in the uppermost focal plane (top guard zone, 5 *μ*m), were exhaustively counted, and the total number of BrdU-labeled cells in the entire SGZ and GCL was estimated.

### 2.12. Fluorescent Microscopy of Mouse Brain Tissues

Free-floating sections were blocked in 2× Block Ace solution for 2 h at room temperature and then incubated with rabbit anti-Iba1 (1 : 500, 019-19741; FUJIFILM Wako Pure Chemical Corporation), mouse anti-human A*β* 82E1 (1 : 2000, 10323; IBL), and rat anti-CD68 (1 : 500, MCA1957GA; Bio-Rad Laboratories, Inc.) overnight at 4°C. Appropriately labeled secondary antibodies with Alexa Fluor 488, 594, and 633 (1 : 300, A11034, A11032, and A21050; Life Technologies Japan, Ltd.) were then added and incubated for 45 min at room temperature, followed by incubation with DAPI (1 : 5000, D8417; Sigma-Aldrich) for 15 min. The sections were then mounted with coverslips using VECTASHIELD Mounting Medium (H-100; Vector Laboratories).

Images were acquired with a confocal laser scanning microscope (LSM-700; Carl Zeiss Microscopy). Microglia clustered around A*β* plaques were defined as clustering microglia. The number of Iba1-positive microglia within 200 *μ*m of clustering microglia in the hippocampus was measured with a 40x objective on an LSM-700 at 0.5 lm intervals along the *z*-axis. The microglial morphology (cell surface area, volume, and sphericity) was analyzed using surface detection and filament reconstruction algorithms (Imaris 7.0; Bitplane, Zurich, Switzerland), referencing previous reports [41]. The morphological parameters obtained from about 100 microglia in each group using 6 sections per mouse (*n* = 4) were analyzed.

### 2.13. Statistical Analyses

All data except for the qRT-PCR findings are expressed as the mean ± standard error of the mean (SEM). The qRT-PCR data are expressed as the mean ± standard deviation (SD). Statistical significance was assessed using the JMP Pro software program, ver. 15.2 (SAS Institute Japan Ltd., Tokyo, Japan). For comparisons among three genotypes, a one-way ANOVA was performed first, and then, post hoc multiple comparisons were performed with the Tukey-Kramer HSD test or Student's *t*-test. When marginal significance was observed in the ANOVA, Hsu's multiple comparisons with the best (MCB) was performed as a post hoc multiple comparison. When comparing two groups, parametric analyses were performed using Student's *t*-test, while nonparametric analyses were performed using Wilcoxon's rank sum exact test. *p* values of <0.05 were considered to indicate statistical significance.

## 3. Results

### 3.1. Hippocampal Expression of MUTYH Proteins in Human Brains

**Figure 2:**
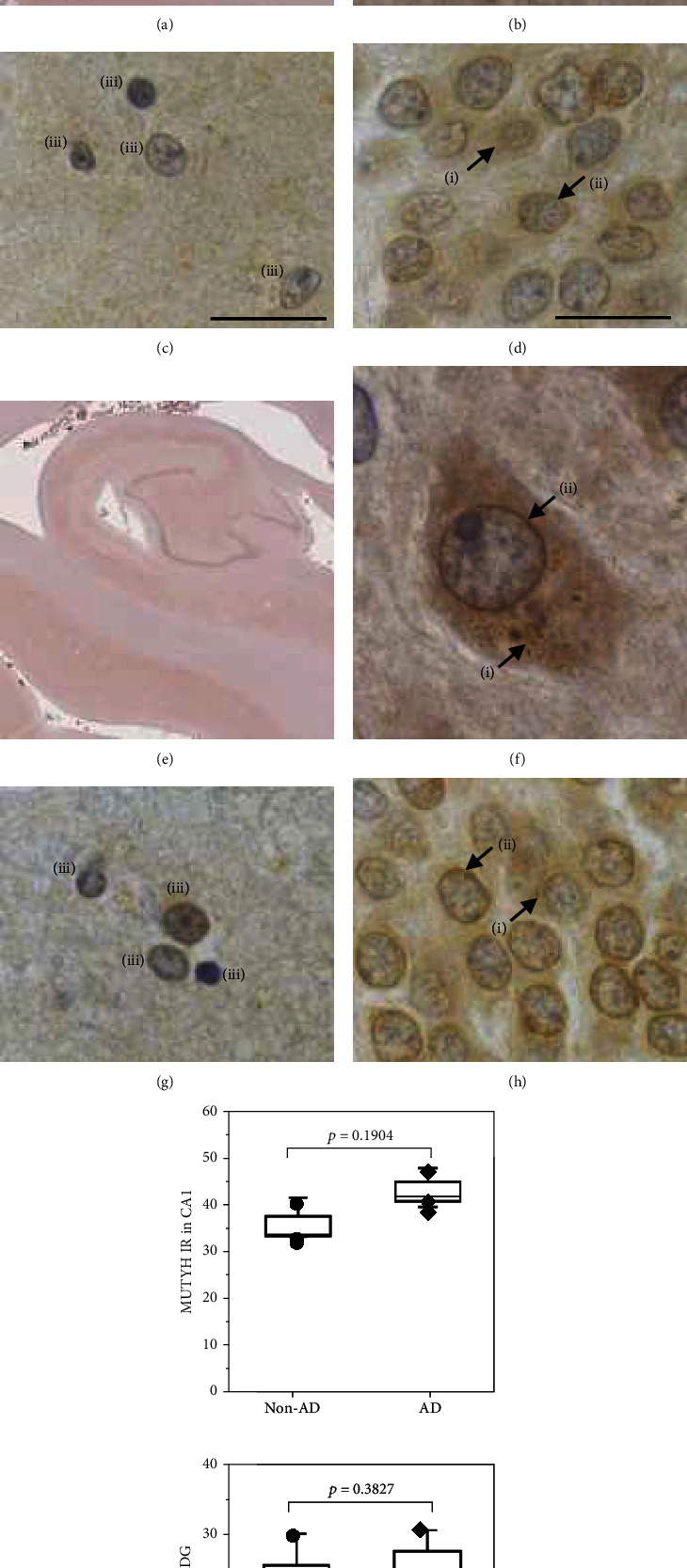
Immunohistochemical detection of MUTYH protein in the human brain. (a–d) Control (non-AD) brain and (e–h) AD brain. (a, e) Low-power view of hippocampal formation and parahippocampal gyrus. MUTYH immunoreactivities were detected in the GCL of the DG and in the pyramidal cell layers of the CA1-CA4 fields and subicular, entorhinal, and perirhinal cortices in both non-AD and AD brains. Nuclei were counterstained by hematoxylin. (b, f) Pyramidal cells in the CA1 field exhibit granular and diffuse MUTYH immunoreactivities in the cytoplasm (i) and to a lesser extent in the nucleus (ii). Intense immunoreactivities were evident in the perinuclear area. (c, g) Glial cells (iii) in the CA1 field exhibit weak MUTYH immunoreactivities in both the nucleus and cytoplasm. (d, h) Granule cells in DG also exhibit granular and diffuse MUTYH immunoreactivities in the cytoplasm (i) and to a lesser extent in the nucleus (ii). Scale bars: 1 mm (a, e) and 20 *μ*m (b–d, f–h). (i) Intensity of MUTYH immunoreactivities (MUTYH IR) in the CA1 field and DG. The data are shown as boxplots with dots and whiskers indicating the minimum and maximum (*n* = 3). Statistical analyses were performed with Wilcoxon's rank sum test, and the *p* value is shown in each graph. Similar levels of MUTYH immunoreactivities were detected in both non-AD and AD hippocampi.

In the control brains without AD pathology (non-AD), MUTYH immunoreactivities were detected in the GCL of the dentate gyrus (DG) and in the pyramidal cell layers of the CA1-CA4 fields and subicular, entorhinal, and perirhinal cortices (Figure 2(a)). Granular and diffuse MUTYH immunoreactivities were detected in both the cytoplasm and perinuclear areas and to a lesser extent in the nuclei of almost all pyramidal and granule cells and glial cells in the hippocampus (Figures 2(b)–2(d), Supplementary Figure S1). In the AD brain, the patterns of MUTYH immunoreactivities were similar to those seen in the non-AD hippocampus and adjacent temporal cortex (Figures 2(e)–2(h)). The signal intensity of MUTYH was slightly higher in both the CA1 field and DG of AD brains than in those of non-AD brains (Figure 2(i)), and the perinuclear MUTYH immunoreactivities in AD cases were markedly higher than those in the non-AD cases (Figures 2(f)–2(h)).

**Figure 3:**
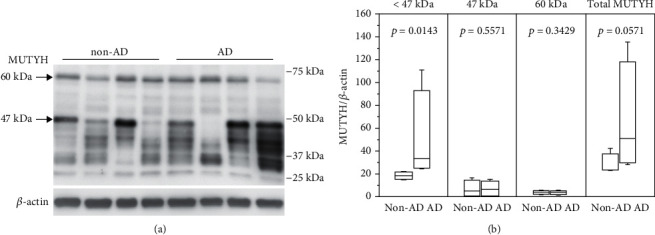
Multiforms of MUTYH protein detected in the human brain. (a) Western blot detection of MUTYH proteins in non-AD and AD hippocampal extracts. (b) A quantitative analysis of the 2 major bands (60 and 47 kDa) and multiple bands of MUTYH protein with molecular weights lower than 47 kDa in non-AD and AD hippocampal extracts. The data were normalized by the *β*-actin levels. *n* = 4. Statistical analyses were performed with Wilcoxon's rank sum test, and the *p* value is shown in each graph. The levels of multiple bands of MUTYH protein with molecular weights lower than 47 kDa were significantly increased in the AD hippocampal extracts.

In human cells, transcription of *MUTYH* is known to be initiated from three distinct exon 1 sequences, thus generating three types of primary transcripts: *α*, *β*, and *γ*. Alternative splicing of the three primary transcripts generates over 10 transcript variants, thereby producing multiple translation products [19, 42, 43]. To examine the expression of multiforms of MUTYH protein in the hippocampus, we performed Western blotting using hippocampal extracts prepared from four non-AD and AD subjects. Two major bands corresponding to 47 and 60 kDa polypeptides were detected (Figure 3(a)). The 60 kDa band was suspected to potentially correspond to the full-length translation products of *MUTYH α*1–3,5 transcripts with the mitochondrial localization signal, *MUTYH β*1–3,5 transcripts, or *MUTYH γ*1–3 transcripts without the mitochondrial localization signal, while the 47 kDa band was deemed likely to correspond to the translation product from *MUTYH α*4, *β*4, or *γ*4 transcripts [19, 42]. There were no significant differences in their expression between non-AD and AD subjects (Figure 3(b)). Multiple bands with molecular weights lower than 47 kDa, suspected to potentially be products of other *MUTYH* transcripts or degradation products of the major bands, were also detected in both non-AD and AD subjects. Compared with the non-AD subjects, these levels were significantly increased in the AD subjects, and even the total levels of MUTYH protein exhibited a strong increasing trend in the AD subjects (Figure 3(b)).

### 3.2. Hippocampal Expression of Multiforms of *MUTYH* Transcripts in Human Brains

We next performed a deep sequencing analysis of RNA prepared from non-AD and AD hippocampi. The expression profile of *MUTYH* transcripts revealed that 15 known *MUTYH* transcript variants, including 2 noncoding RNAs, are expressed in the human hippocampus (Table 1, Supplementary Table S2).

According to the first exon sequences and alternative splicing patterns of exon 3, we confirmed that *MUTYH* transcripts corresponding to types *α*1 to *α*5, *β*1, *β*3, *β*5, and *γ*2, *γ*3, and *γ*4, which are known to encode various MUTYH isoforms corresponding to the major 60 and 47 kDa MUTYH polypeptides detected in Figure 3(a), are indeed expressed in the human hippocampus. We also detected variant 14 (NR_146882.2) and variant 15 (NR_146883.2), which are considered to be noncoding; however, these variants may encode 52.7 and 39.6 kDa polypeptides, respectively. We further identified six novel transcript variants (MSTRG.709.x in Table 1, Supplementary Table S2) carrying altered exons and/or retained introns in both non-AD and AD samples. MSTRG.709.1, MSTGR.709.2, and MSTRG.709.4, all of which have retained intron 5, may encode identical 37.8 kDa polypeptides, while MSTRG.709.3, which carries a novel first exon and has retained introns 4, 5, and 8, may encode a 29.4 kDa polypeptide. MSTRG.709.20, which has retained introns 4 and 11 and a shortened exon 16, and MSTRG.709.21, which starts at exon 3 with retained intron 11 and shortened exon 16, are expected to be noncoding.

The expression of *MUTYH* at the gene level was significantly higher in AD subjects than in non-AD subjects (log_2_[fold change] [logFC] = 0.614, likelihood ratio [LR] = 7.929, *p* value = 0.005, and false discovery rate [FDR] = 0.103); however, there is no significant difference in expression levels of each transcript variant between non-AD and AD cases. We confirmed the expression of the multiforms of *MUTYH* transcripts in the hippocampus by RT-PCR with three sets of type-specific primers, and most of the major and minor bands corresponding to isoforms identified by RNA sequencing were amplified in both non-AD and AD samples (Supplementary Figure S2).

Taken together, these results indicate that multiforms of *MUTYH* transcripts are indeed expressed in the human hippocampus, in both non-AD and AD subjects, with the latter exhibiting an increased expression of the *MUTYH* gene, thereby contributing to the increased levels of multiforms of MUTYH protein in the AD subjects.

### 3.3. Hippocampal Accumulation of 8-oxoG in Human Brains

**Figure 4:**
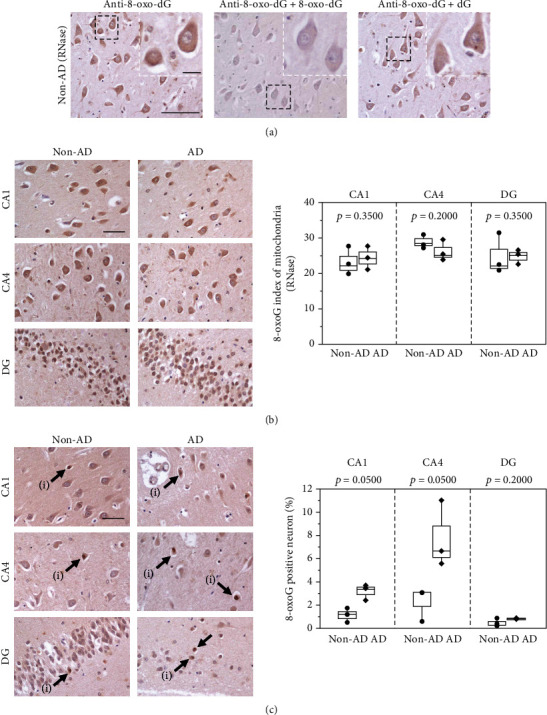
Immunohistochemical detection of 8-oxoG in human brains. (a) Validation of 8-oxoG immunoreactivity detected by the anti-8-oxo-dG antibody. Sections of the non-AD hippocampus pretreated only with RNase were subjected to immunohistochemistry with the anti-8-oxo-dG antibody and the antibody preadsorbed by 8-oxo-dG (anti-8-oxo-dG + 8-oxo-dG) or 2′-deoxyguanosine (anti-8-oxo-dG + dG). Cytoplasmic immunoactivities were observed in the neurons of the non-AD hippocampus (left). Preadsorption of the antibody with 8-oxo-dG (middle) but not with dG (right) abolished the immunoactivity. Scale bar = 100 *μ*m for the full image and 20 *μ*m for magnified images. (b) Immunohistochemical detection and quantification of 8-oxoG in mitochondrial DNA of the hippocampus. The sections were pretreated only with RNase. (c) Immunohistochemical detection and quantification of 8-oxoG in nuclear DNA of the CA1 field (CA1), CA4 field (CA4), and DG. The sections were pretreated with RNase followed by HCl. Nuclear 8-oxoG immunoreactivity-positive neuron (i). Scale bars = 50 *μ*m. The data are shown as boxplots with dots and whiskers indicating the minimum and maximum (*n* = 3). Statistical analyses were performed with Wilcoxon's rank sum test, and the *p* value is shown in each graph.

To examine the 8-oxoG levels that had accumulated in human brains, we performed the immunohistochemical detection of 8-oxoG in the non-AD and AD hippocampus (Figure 4). As AD brains reportedly exhibit a significantly increased 8-oxoG accumulation in RNA [4], pretreatment with RNase was performed to avoid detecting 8-oxoG in RNA, and the remaining immunoreactivities were abolished by preadsorbing the antibody with 8-oxo-dG but not dG (Figure 4(a)), thus confirming the specificity of the antibody.

To distinguish 8-oxoG that had accumulated in the mitochondrial and nuclear DNA, we applied the anti-8-oxo-dG antibody to sections with or without HCl pretreatment, which denatures chromatin structure and depletes mitochondrial DNA [44], thereby enabling the selective detection of 8-oxoG in nuclear DNA (Figures 4(b) and 4(c)). In the absence of HCl pretreatment, 8-oxoG immunoreactivity was detected in the cytoplasm, indicating 8-oxoG in mitochondrial DNA, and extremely high levels of the 8-oxoG index were observed in the mitochondrial DNA from both the non-AD and AD subjects, with no significant difference noted (Figure 4(b)). In contrast, with HCl pretreatment, 8-oxoG immunoreactivities were mainly detected in the nuclei of neurons, and the proportion of 8-oxoG-positive neurons was significantly increased in the CA1 and CA4 fields but not the GCL of DG in the AD subjects compared to the non-AD subjects (Figure 4(c)).

These results indicate that 8-oxoG is strongly accumulated in the mitochondrial DNA of hippocampal neurons, regardless of the AD pathology, in the human brain, but neurons in the CA1 and CA4 fields in the AD brain show an increased 8-oxoG accumulation in nuclear DNA.

### 3.4. MUTYH Deficiency Attenuates the Moderate Cognitive Impairment in *App*^NL-G-F/NL-G-F^ Mice

**Figure 5:**
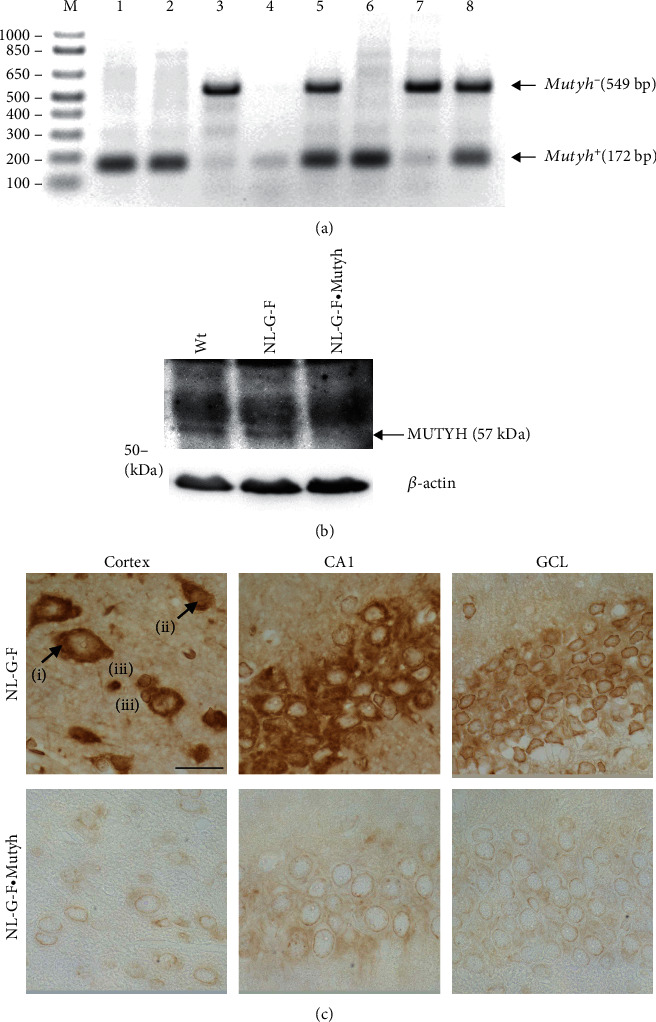
The expression of MUTYH protein in wild-type, *App*^NL-G-F/NL-G-F^, and *App*^NL-G-F/NL-G-F^·*Mutyh*^−/−^ mouse brains. (a) Genotyping of wild-type, *App*^NL-G-F/NL-G-F^, and *App*^NL-G-F/NL-G-F^·*Mutyh*^−/−^ mice using tail DNA. *Mutyh*^+^ and *Mutyh*^−^ alleles were amplified using specific primer sets. Lane 1, wild type; 2 and 6, *App*^NL-G-F/NL-G-F^; 3 and 7, *App*^NL-G-F/NL-G-F^·*Mutyh*^−/−^; 4, no tail DNA (negative control); and 5 and 8, *Mutyh*^+/-^ (positive control). (b) Hippocampal extracts prepared from six-month-old wild-type (Wt), *App*^NL-G-F/NL-G-F^ (NL-G-F), and *App*^NL-G-F/NL-G-F^·*Mutyh*^−/−^ (NL-G-F·Mutyh) mice were subjected to Western blotting with the anti-MUTYH antibody. *β*-Actin was detected as a loading control. (c) The immunohistochemical detection of MUTYH protein in *App*^NL-G-F/NL-G-F^ (NL-G-F) and *App*^NL-G-F/NL-G-F^·*Mutyh*^−/−^ (NL-G-F·Mutyh) mouse brains. The signal was predominantly detected in cortical (cortex), pyramidal (CA1), and granular (GCL) neurons. Cytoplasmic (i) and nuclear (ii) immunoreactivities of MUTYH were detected in the neurons of the cortex. MUTYH was also expressed in glial cells (iii). Scale bar = 20 *μ*m.

The increased expression of MUTYH in the AD brains may suggest that MUTYH actively contributes to the AD pathogenesis. To clarify whether or not MUTYH is involved in AD pathogenesis, we examined the effect of MUTYH deficiency on an *App* knock-in AD model, *App*^NL-G-F/NL-G-F^ mice [28]. *App*^NL-G-F/NL-G-F^ mice exhibited the progressive accumulation of A*β* starting at four to six months of age, dense distributions of activated microglia and astrocytes from five to seven months of age, and behavioral symptoms from six months of age but lacked NFTs and neuronal loss, reflecting the features of preclinical AD [28, 34, 45]. We then confirmed that brains of *App*^NL-G-F/NL-G-F^·*Mutyh*^−/−^ mice (Figure 5(a)) were indeed deficient in MUTYH protein by Western blotting (Figure 5(b)) and immunohistochemistry (Figure 5(c)) for MUTYH protein. In the mouse brain, granular and diffuse MUTYH immunoreactivities were detected in both the cytoplasm and perinuclear areas and to a lesser extent in the nuclei of neurons and glial cells (Figure 5(c)).

We monitored the spontaneous locomotor activity in the home cage and performed an open-field test and novel objective recognition test using six- and twelve-month-old male and female wild-type, *App*^NL-G-F/NL-G-F^, and *App*^NL-G-F/NL-G-F^·*Mutyh*^−/−^ mice (*n* = 13-15 per group) (Figure 1(a)).

At six months of age, the locomotor activity, especially in the dark phase, was increased only in female *App*^NL-G-F/NL-G-F^ mice compared with wild-type mice (Figure 1(c); Hsu's MCB, *p* = 0.02; Supplementary Figure S3f). At twelve months of age, the locomotor activities in the dark phase were decreased in all groups compared to values at six months of age, except in female *App*^NL-G-F/NL-G-F^·*Mutyh*^−/−^ mice (Figures 1(c) and 1(e)), Supplementary Figure S3), and only female *App*^NL-G-F/NL-G-F^ and *App*^NL-G-F/NL-G-F^·*Mutyh*^−/−^ mice exhibited significantly higher activities than wild-type mice (Figure 1(e)).

In the open-field test, male *App*^NL-G-F/NL-G-F^·*Mutyh*^−/−^ mice exhibited a significantly longer total travel distance than wild-type mice (Figure 1(f)); essentially, the same difference was observed with regard to supportive rearing (Supplementary Figure S4a). In contrast, at twelve months of age, male but not female *App*^NL-G-F/NL-G-F^ and *App*^NL-G-F/NL-G-F^·*Mutyh*^−/−^ mice exhibited a significantly shorter total travel distance than wild-type mice (Figure 1(h)). Neither male nor female mice exhibited any significant difference in time spent in the center or at the periphery at six or twelve months of age (Supplementary Figure S4e-l).

In the novel objective recognition test, male—and to a lesser extent female—*App*^NL-G-F/NL-G-F^ mice showed a markedly reduced capacity to recognize novel objects, while *App*^NL-G-F/NL-G-F^·*Mutyh*^−/−^ mice exhibited a recovered capacity to recognize novel objects (Figures 1(j) and 1(k)), Supplementary Figure S5a, b). Twelve-month-old mice exhibited no significant difference in their capacity to recognize novel objects (Figures 1(l) and 1(m)), Supplementary Figure S5c, d).

These results indicated that *App*^NL-G-F/NL-G-F^ mice exhibit a moderately impaired cognitive function with some sex- and age-related behavioral alterations, especially at six months of age, and MUTYH deficiency improved the impairment.

### 3.5. MUTYH Deficiency Alters Neither Amyloid-*β* Formation Nor Deposition in the *App*^NL-G-F/NL-G-F^ Brain

**Figure 6:**
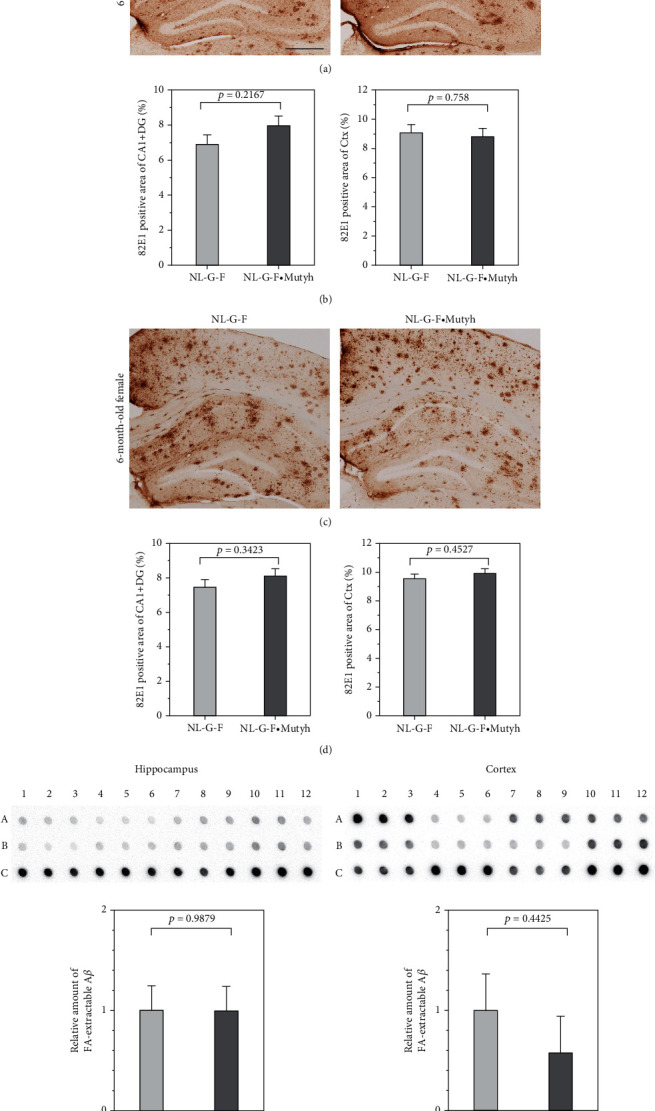
The A*β* deposition in the *App*^NL-G-F/NL-G-F^ brain was not changed by MUTYH deficiency. (a–d) Immunohistochemistry with anti-A*β* (82E1) in the six-month-old male (a) and female (c) brain. *App*^NL-G-F/NL-G-F^ (NL-G-F) and *App*^NL-G-F/NL-G-F^·*Mutyh*^−/−^ (NL-G-F·Mutyh). Scale bar = 500 *μ*m. (b, d) The A*β*-positive area in the hippocampus, including the CA1 field and DG (CA1 + DG) and cortex (Ctx), was measured in every 5 coronal sections (4 sections per mouse; bregma -1.555 to -2.155 mm). Statistical analyses were performed with Student's *t*-test, and the *p* value is shown in each bar graph (mean ± SEM, *n* = 4). (e, f) The results of a dot blot analysis of SDS-insoluble/formic acid (FA)-extractable A*β* in the six-month-old female mouse hippocampus (e) and cortex (f) with anti-A*β* (6E10) are shown. Top panels: A1 to A12, six-month-old female *App*^NL-G-F/NL-G-F^ samples; B1 to B12, six-month-old female *App*^NL-G-F/NL-G-F^ ·*Mutyh*^−/−^ samples; and C1 to C3, twelve-month-old female *App*^NL-G-F/NL-G-F^ samples as a control. Bottom panels: the intensity of SDS-insoluble/FA-extractable A*β* was normalized to the brain weight, and the values are relative to the average intensity of six-month-old female *App*^NL-G-F/NL-G-F^ samples. Statistical analyses were performed with Student's *t*-test, and the *p* value is shown in each bar graph (mean ± SEM, *n* = 4).

To evaluate the effects of MUTYH deficiency on A*β* deposition, we performed immunohistochemistry with the anti-A*β* (82E1) antibody and calculated the area of A*β* deposition (Figures 6(a)–6(d)). In six-month-old male and female mice, the area of A*β* deposition in the hippocampus (CA1 field and DG) and cortex did not differ markedly between *App*^NL-G-F/NL-G-F^ and *App*^NL-G-F/NL-G-F^·*Mutyh*^−/−^ mice (Figures 6(b) and 6(d)). The SDS-insoluble form of A*β* was further extracted using formic acid (FA) and then subjected to a dot blot analysis (Figures 6(e) and 6(f)). The quantitative measurement of the dot blots revealed that the levels of the SDS-insoluble/FA-extractable form of A*β* in the six-month-old female brain were not markedly different between *App*^NL-G-F/NL-G-F^ and *App*^NL-G-F/NL-G-F^·*Mutyh*^−/−^ mice. We also performed a Western blotting analysis of the SDS-soluble form of A*β* in six-month-old female hippocampal extracts and confirmed that the levels of the SDS-soluble form of A*β* were not markedly different between *App*^NL-G-F/NL-G-F^ and *App*^NL-G-F/NL-G-F^·*Mutyh*^−/−^ mice (Supplementary Figure S6).

These results indicate that MUTYH deficiency did not alter A*β* formation or deposition in the six-month-old *App*^NL-G-F/NL-G-F^ mouse brain.

### 3.6. MUTYH Deficiency Significantly Alters the Expression Profiles of Microglia in the *App*^NL-G-F/NL-G-F^ Hippocampus

We performed a microarray analysis using RNA prepared from six-month-old female wild-type, *App*^NL-G-F/NL-G-F^, and *App*^NL-G-F/NL-G-F^·*Mutyh*^−/−^ hippocampi and compared the expression of genes encoding specific markers for four major types of brain cells (neurons, astrocytes, oligodendrocytes, and microglia) to evaluate whether or not MUTYH deficiency alters cell populations (Table 2).

The expression of markers for neural stem and progenitor cell (NSC/NPC) [46–48] and markers for mature neurons, including *Rbfox3* encoding neuronal nuclear antigen (NeuN), was not markedly altered in *App*^NL-G-F/NL-G-F^ and *App*^NL-G-F/NL-G-F^·*Mutyh*^−/−^ mice compared with wild-type mice. These results suggest that there is not much neuronal damage or loss in *App*^NL-G-F/NL-G-F^ and *App*^NL-G-F/NL-G-F^·*Mutyh*^−/−^ hippocampi.

The relative expression of *Gfap* was increased in *App*^NL-G-F/NL-G-F^ and *App*^NL-G-F/NL-G-F^·*Mutyh*^−/−^ mice, although other astrocyte markers were roughly the same as those in wild-type mice. We then investigated more markers for Pan-reactive astrocytes, A1 astrocytes, and A2 astrocytes [49, 50] (Supplementary Table S3). The mean relative expression of the three types of markers in *App*^NL-G-F/NL-G-F^ and *App*^NL-G-F/NL-G-F^·*Mutyh*^−/−^ mice was not significantly different from that in wild-type mice. The expression of most oligodendrocyte markers was slightly decreased in *App*^NL-G-F/NL-G-F^ mice and to a greater extent in *App*^NL-G-F/NL-G-F^·*Mutyh*^−/−^ mice (Table 2).

The expression of several microglial markers related to activation or inflammation states was increased in the *App*^NL-G-F/NL-G-F^ hippocampus, and among them, the expression of *Cd68* encoding lysosomal protein was most significantly increased in comparison to that in wild-type mice. In contrast, the expression of *Cd68* and *Lgals3* was markedly decreased in the *App*^NL-G-F/NL-G-F^·*Mutyh*^−/−^ hippocampus compared with the *App*^NL-G-F/NL-G-F^ hippocampus, suggesting that MUTYH deficiency selectively attenuates microglial activation caused by the pathogenic A*β* accumulation (Table 2).

It was recently shown that novel disease-associated microglia (DAM) identified in AD model mice are induced from homeostatic microglia through two stages (stage 1 and stage 2 DAM) [51, 52], with unique gene expression profiles. We therefore compared the expression of key genes for homeostatic microglia and stage 1 and 2 DAM in wild-type, *App*^NL-G-F/NL-G-F^, and *App*^NL-G-F/NL-G-F^·*Mutyh^−/−^* hippocampi (Table 3). The expression of 8 out of 12 homeostatic genes was significantly increased in the *App*^NL-G-F/NL-G-F^ hippocampus compared with the wild-type hippocampus, and the expression of 7 of these genes remained significantly higher in the *App*^NL-G-F/NL-G-F^·*Mutyh^−/−^* hippocampus than in the wild-type hippocampus. Only one gene (*Sparc*) showed a significantly decreased expression in the *App*^NL-G-F/NL-G-F^·*Mutyh^−/−^* hippocampus compared with the *App*^NL-G-F/NL-G-F^ hippocampus, with the value falling below even the wild-type level. Among 10 genes evaluated for stage 1 DAM, 4 (*Tyrobp*, *Ctsd*, *Lyz2*, and *B2m*) showed a significantly increased expression in the *App*^NL-G-F/NL-G-F^ hippocampus compared with the wild-type hippocampus, while 3 (*Ctsd*, *Lyz2*, and *Timp2*) were significantly downregulated in the *App*^NL-G-F/NL-G-F^·*Mutyh^−/−^* hippocampus compared with the *App*^NL-G-F/NL-G-F^ hippocampus. Eleven out of 21 genes for stage 2 DAM were significantly upregulated in the *App*^NL-G-F/NL-G-F^ hippocampus compared with the wild-type hippocampus, while the expression of 4 (*Cd68*, *Cst7*, *Trem*2, and *Ccl6*) was significantly downregulated in the *App*^NL-G-F/NL-G-F^·*Mutyh^−/−^* hippocampus compared with the *App*^NL-G-F/NL-G-F^ hippocampus.

Furthermore, based on ANOVA (eBayes), we identified 138 transcription clusters whose expression in *App*^NL-G-F/NL-G-F^ mice was significantly different from that in wild-type or *App*^NL-G-F/NL-G-F^·*Mutyh^−/−^* mice and analyzed these clusters using the functional annotation clustering tools in DAVID. A total of 103 genes were found to be functionally annotated (Supplementary Table S4). The top annotation cluster 1 with the term “lysozyme” exhibited the highest enrichment and included seven genes (*Npc2*, *Psap*, *Gpc3*, *Cd68*, *Ctsd*, *Rnaset2b*, and *Rnaset2a*) that were likely to be involved in phagocytosis by microglia; all of them were downregulated by MUTYH deficiency (Table 4). The second annotation cluster 2 mainly contained secreted extracellular proteins, and some of them were expressed in DAM (Tables 3 and 4).

Taken together, these results strongly suggest that phagocytic DAM are highly activated in the *App*^NL-G-F/NL-G-F^ hippocampus, and MUTYH deficiency significantly attenuates this activation.

### 3.7. MUTYH Deficiency Attenuates Microglial Activation in the *App*^NL-G-F/NL-G-F^ Hippocampus

**Figure 7:**
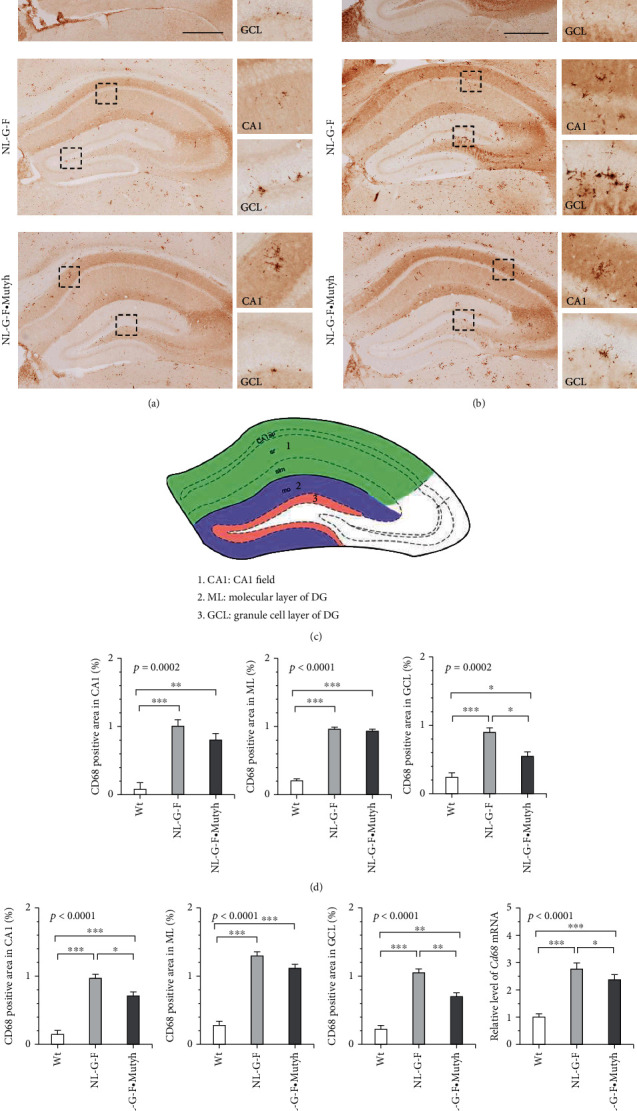
MUTYH deficiency attenuated microgliosis in the *App*^NL-G-F/NL-G-F^ brain. (a, b) Immunohistochemistry for CD68 in six-month-old male (a) and female (b) brains. Scale bar = 500 *μ*m for the full image and 50 *μ*m for magnified images. Wild-type (Wt), *App*^NL-G-F/NL-G-F^ (NL-G-F), and *App*^NL-G-F/NL-G-F^·*Mutyh*^−/−^ (NL-G-F·Mutyh). (c) Diagram depicting the three studied zones in the hippocampus: the CA1 field, ML, and GCL of DG. (d, e) The CD68-positive area in the CA1 field, ML, and GCL of DG was measured in every 5 coronal sections (4 sections per mouse; bregma -1.555 to -2.155 mm) prepared from six-month-old male (d) and female (e) brains, and the results are shown as the mean ± SEM (*n* = 4). (f) Female hippocampal levels of *Cd68* mRNA determined by qRT-PCR. The data were normalized by the levels of *Gapdh* mRNA and expressed as the mean ± SD (*n* = 4). Statistical analyses were performed with a one-way ANOVA (the *p* value is shown in each bar graph) followed by a post hoc Tukey-Kramer HSD test. ^∗^*p* < 0.05, ^∗∗^*p* < 0.005, and ^∗∗∗^*p* < 0.0005.

To compare the microglial status among wild-type, *App*^NL-G-F/NL-G-F^, and *App*^NL-G-F/NL-G-F^·*Mutyh*^−/−^ hippocampi, we performed immunohistochemistry for CD68, the expression of which was markedly changed in a microarray analysis (Figures 7(a) and 7(b)). In the six-month-old male and female *App*^NL-G-F/NL-G-F^ hippocampus, including the CA1 field, the molecular layer (ML), and the GCL of DG, the CD68-positive area was significantly increased compared to that in the wild-type hippocampus (Figures 7(d) and 7(e)). Among the three zones in the male *App*^NL-G-F/NL-G-F^·*Mutyh*^−/−^ hippocampus, only the GCL exhibited a significantly decreased CD68-positive area compared with the *App*^NL-G-F/NL-G-F^ hippocampus (Figures 7(a) and 7(d)). In the female *App*^NL-G-F/NL-G-F^·*Mutyh*^−/−^ hippocampus, the CD68-positive area in the GCL but not the ML of the DG, and to a lesser extent the CA1 field, was significantly decreased compared with the *App*^NL-G-F/NL-G-F^ hippocampus (Figures 7(b) and 7(e)). The female hippocampal levels of *Cd68* mRNA, as determined by qRT-PCR, confirmed the results of CD68 immunochemistry as well as the results of the microarray analysis (Figure 7(f)). These results suggest that activated microglia in the GCL may play an important role in the impairment of the cognitive function.

**Figure 8:**
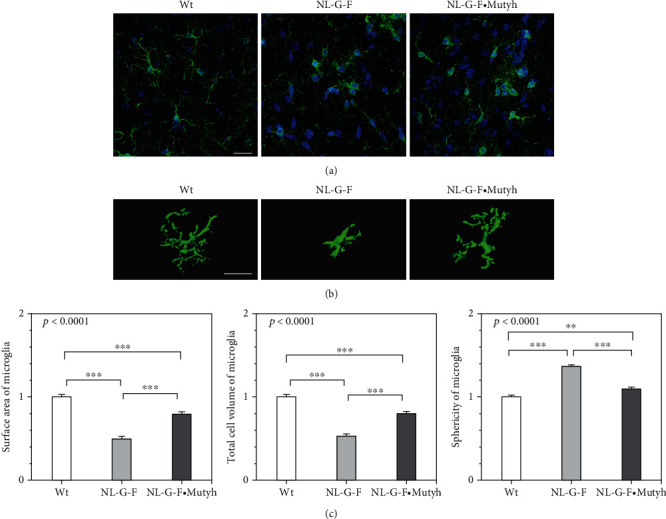
MUTYH deficiency suppressed the morphological alteration of microglia in the *App*^NL-G-F/NL-G-F^ brain. (a) Immunofluorescent micrograph of hippocampal microglia stained for Iba-1 (green) in six-month-old female wild-type (Wt), *App*^NL-G-F/NL-G-F^ (NL-G-F), and *App*^NL-G-F/NL-G-F^·*Mutyh*^−/−^ (NL-G-F·Mutyh) mice. Nuclei were counterstained with DAPI (blue). Scale bar = 100 *μ*m. (b) Three-dimensional reconstruction of microglia surrounding A*β* plaque in the six-month-old female hippocampus. Scale bar = 100 *μ*m. (c) Morphological parameters of hippocampal microglia (cell surface area, total cell volume, and sphericity). Each parameter was normalized by the wild-type value. The number of microglia examined was 101 to 107 in each group. The data are expressed as the mean ± SEM (*n* = 4). Statistical analyses were performed with a one-way ANOVA (*p* value shown in each bar graph) followed by the post hoc Tukey-Kramer HSD test. ^∗∗^*p* < 0.005 and ^∗∗∗^*p* < 0.0005.

We showed that microglia were highly clustered inside A*β* plaques (Supplementary Figure S7a) and that CD68-positive microglia were largely observed inside A*β* plaques in the *App*^NL-G-F/NL-G-F^ hippocampus (Supplementary Figure S7b). To further address whether or not MUTYH deficiency causes the morphological alteration of microglia, wild-type, *App*^NL-G-F/NL-G-F^, and *App*^NL-G-F/NL-G-F^·*Mutyh*^−/−^ hippocampal microglia detected by immunofluorescent microscopy for Iba-1 were subjected to a detailed three-dimensional (3D) morphological analysis. In the six-month-old female *App*^NL-G-F/NL-G-F^ mouse hippocampus, microglia clustered and exhibited an amoeboid-shaped round cell body with a few ramified processes (Figure 8(a)). An analysis of 3D-reconstructed microglia in the *App*^NL-G-F/NL-G-F^ CA1 field revealed a significantly reduced surface and volume and significantly increased sphericity index compared with the wild-type hippocampus, confirming that microglia were highly activated (Figures 8(b) and 8(c)). In contrast, microglia in the *App*^NL-G-F/NL-G-F^·*Mutyh*^−/−^ hippocampus exhibited a ramified phenotype similar to the wild-type hippocampus, thus confirming that MUTYH deficiency significantly attenuates microglial activation (Figures 8(b) and 8(c)).

### 3.8. MUTYH Deficiency Decreased the Accumulation of 8-oxoG in *App*^NL-G-F/NL-G-F^ Mouse Brains

**Figure 9:**
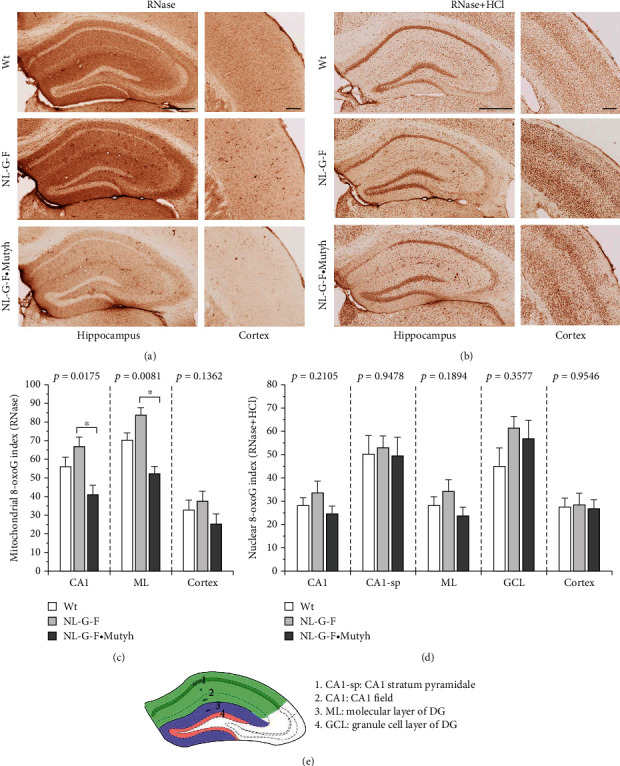
MUTYH deficiency decreased the accumulation of 8-oxoG in six-month-old female *App*^NL-G-F/NL-G-F^ mouse brains. (a) Immunohistochemical detection of 8-oxoG in mitochondrial DNA with RNase pretreatment. (b) Immunohistochemical detection of 8-oxoG in nuclear DNA with RNase and HCl pretreatment. Scale bar = 500 *μ*m for the hippocampus and 200 *μ*m for the cortex. Wild-type (Wt), *App*^NL-G-F/NL-G-F^ (NL-G-F), and *App*^NL-G-F/NL-G-F^·*Mutyh*^−/−^ (NL-G-F·Mutyh). (c, d) Quantitative measurement of mitochondrial (c) and nuclear (d) 8-oxoG in the brain. (e) Diagram depicting the four studied zones in the hippocampus: CA1-sp, entire CA1 field (CA1), ML, and GCL of DG. These fields were measured in every 5 coronal sections (4 sections per mouse; bregma -1.555 to -2.155 mm), and the results are shown as the mean ± SEM (*n* = 4). Statistical analyses were performed with a one-way ANOVA (*p* value shown in each bar graph), followed by the post hoc Tukey-Kramer HSD test. ^∗^*p* < 0.05.

To evaluate the effects of MUTYH deficiency on the accumulation of 8-oxoG in the *App*^NL-G-F/NL-G-F^ brain, we performed the immunohistochemical detection of 8-oxoG in six-month-old female mouse brains (Figure 9). With RNase pretreatment, substantial levels of 8-oxoG in mitochondrial DNA were detected throughout the entire hippocampus, except for the pyramidal cell layer in the CA1 (CA1-Sp) to CA3 fields and GCL, and to a lesser extent in the cortex of wild-type mice, and the levels were slightly increased in *App*^NL-G-F/NL-G-F^ brains. MUTYH deficiency significantly decreased the 8-oxoG levels in the entire CA1 field and ML of DG in the hippocampus and to a lesser extent in cortex compared to *App*^NL-G-F/NL-G-F^ brains, the values of which were even lower than those in wild-type mice (Figures 9(a) and 9(c)).

With the pretreatment of RNase and HCl, high levels of 8-oxoG in nuclear DNA were detected mostly in the CA1-Sp and GCL and to a lesser extent in the cortex of wild-type mice, and the levels were also slightly increased in the hippocampus, especially in the GCL, but not in the cortex of *App*^NL-G-F/NL-G-F^ mice. *App*^NL-G-F/NL-G-F^·*Mutyh*^−/−^ mice slightly decreased the 8-oxoG levels in the hippocampus compared to *App*^NL-G-F/NL-G-F^ mice, albeit without statistical significance (Figures 9(b) and 9(d)).

These results suggest that MUTYH deficiency may significantly decrease oxidative stress, especially in the hippocampus, likely because of attenuated microgliosis.

### 3.9. MUTYH Deficiency Improves Neurogenesis in the *App*^NL-G-F/NL-G-F^ Hippocampus

**Figure 10:**
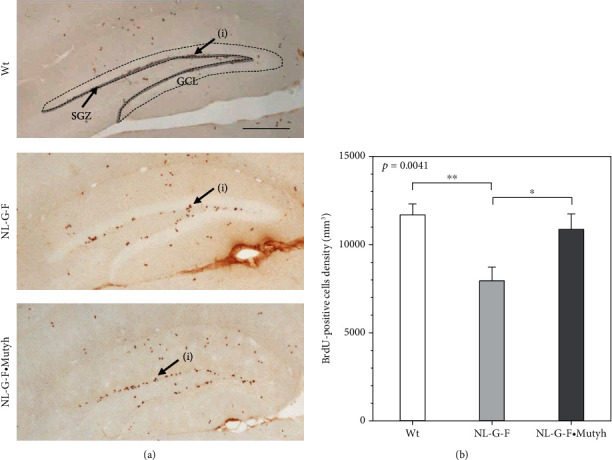
MUTYH deficiency enhances neurogenesis. (a) Immunohistochemical detection of BrdU-positive cells (i) in the neurogenic niche, SGZ (gray area), and GCL (area surrounded by the dotted line). Scale bar = 200 *μ*m. (b) Density of BrdU-positive cells in the neurogenic niche. Measured sections were placed 200 *μ*m away from each other (11 sections per mouse; bregma -1.350 to -3.510 mm), and the results are shown as the density of BrdU-positive cells in the GCL + SGZ (numbers of cells/mm^3^) in a bar graph (mean ± SEM, *n* = 4-5). Statistical analyses were performed with a one-way ANOVA (*p* value shown in each bar graph) followed by the post hoc Tukey-Kramer HSD test. ^∗^*p* < 0.05 and ^∗∗^*p* < 0.005.

A small number of newborn neurons originating in the SGZ migrate to the GCL, thereby contributing to the maintenance of the cognitive function in the hippocampus, and AD model mice exhibit decreased neurogenesis [53, 54]. Therefore, to investigate neurogenesis in the hippocampus, we labeled newborn neurons in six-month female wild-type, *App*^NL-G-F/NL-G-F^, and *App*^NL-G-F/NL-G-F^·*Mutyh*^−/−^ mouse brains with BrdU (Figure 10(a)). Twenty-four hours after the last injection of BrdU, the density of BrdU-positive cells in the SGZ and GCL was significantly decreased in the *App*^NL-G-F/NL-G-F^ brain compared with the wild-type brain (Figure 10(b)). We further examined the effects of MUTYH deficiency on neurogenesis and found that the density of BrdU-positive cells in the SGZ and GCL in six-month-old female *App*^NL-G-F/NL-G-F^·*Mutyh*^−/−^ mice had recovered to the level seen in wild-type mice (Figure 10(b)).

Our results strongly suggest that MUTYH actively contributes to AD pathogenesis by activating microglia and impairing neurogenesis in the hippocampus, thus resulting in the mild cognitive impairment seen in *App*^NL-G-F/NL-G-F^ mice.

## 4. Discussion

The present study revealed for the first time that multiforms of *MUTYH* mRNA and MUTYH protein are expressed in human hippocampal neurons and glia, regardless of the AD pathology. We also clarified that MUTYH deficiency in *App*^NL-G-F/NL-G-F^ knock-in AD model mice improved behavioral and cognitive impairments and significantly decreased microgliosis. Furthermore, MUTYH deficiency improved neurogenesis in the hippocampus, which is markedly impaired in *App*^NL-G-F/NL-G-F^ mice. Taken together, the present study showed that MUTYH plays a detrimental role during AD pathogenesis through excessive microglial activation.

MUTYH has been suggested to actively contribute to the neurodegenerative process in Parkinson's diseases [55] and Huntington disease [56]. *MUTYH* mRNA levels in blood lymphocytes are reportedly decreased in AD patients [57], and some single-nucleotide polymorphisms in the *MUTYH* gene are associated with AD [58]; however, no studies have reported the direct observation of how MUTYH is expressed in the human AD brain. We demonstrated that in both the normal and AD human brain, especially in the hippocampus, the pyramidal, granule, and glial cells express substantial levels of MUTYH protein, which is localized in the cytoplasm and perinuclear areas and to a lesser extent in the nuclei. In AD cases, MUTYH immunoreactivities in the perinuclear areas are markedly higher than those in non-AD cases. As lamin A localized in the perinuclear area has been shown to promote the base excision repair (BER) of 8-oxoG [59] and perinuclear lamin A, which is not typically expressed in neurons, has been detected at the transformation from senile to AD hippocampal neurons [60], it is likely that MUTYH protein is preferentially localized in the perinuclear area of AD neurons and thus initiates BER of adenine opposite 8-oxoG in DNA.

Some isoforms of MUTYH protein carry amino-terminal mitochondrial targeting signals (MTSs) as well as nuclear localization signals (NLSs) [19, 42]. We detected in the hippocampus the expression of type *α*1, *α*2, *α*3, and *α*5 mRNA-encoding isoforms (1, 6, 2, and 5, respectively) carrying both MTSs and two NLSs as well as the expression of type *β*1, *β*3, and *β*5 and *γ*2 and *γ*3 mRNA-encoding isoforms (7, 4, 4, 3, and 4, respectively) carrying only the two NLSs; those isoforms are likely to correspond to the 60 kDa polypeptide detected by Western blotting. We also detected type *α*4 and *γ*4 mRNAs, which encode isoform 8 carrying only the carboxy-terminal NLS (expected to be the 47 kDa polypeptide detected by Western blotting). There are many bands smaller than 47 kDa in size, and some may be translation products from the variant 15 (NR_146883.2) and novel *MUTYH* mRNAs (MSTRG.709.1, MSTRG.709.2, MSTRG.709.3, and MSTRG.709.4) identified in this study, such as the 39.6, 37.8, and 29.4 kDa polypeptides shown in Table 1. In addition, we found that there was no marked difference in the protein isoforms, their intracellular localization, or the cell types expressing MUTYH protein in the AD and non-AD hippocampus. These results suggest that MUTYH protein isoforms are localized and involved in BER of adenine mispaired with 8-oxoG in template DNA in both the mitochondria and nuclei of the human hippocampus.

In the present study, we introduced MUTYH deficiency into *App* knock-in AD model mice carrying homozygous *App*^NL-G-F^ alleles, which produce humanized A*β* without the overexpression of APP protein. *App*^NL-G-F/NL-G-F^ mice reportedly display a relatively mild behavioral phenotype, characteristic of preclinical AD, although there are some differences based on the age, sex, and environment [61–66]. In our study, at six and twelve months of age, both male and female *App*^NL-G-F/NL-G-F^ mice exhibited significant but mild behavioral alternations, including memory impairment, compared to wild-type mice. Importantly, we found that MUTYH deficiency apparently ameliorates only the memory impairment in six-month-old *App*^NL-G-F/NL-G-F^ mice; however, the differences observed in the findings of the open-field test and spontaneous locomotor activity in the home cage were not altered. It has been shown that MUTYH deficiency moderately improves learning and memory in four- to seven-month-old male mice in the Morris water maze test [67, 68]. These present and previous findings indicate that MUTYH deficiency improves the memory function, even with an AD pathology.

A microarray analysis suggested significant microglial activation in the six-month-old female *App*^NL-G-F/NL-G-F^ hippocampus, with the *Cd68* mRNA expression being markedly higher than that in the wild-type hippocampus. Interestingly, the *Cd68* mRNA expression in the *App*^NL-G-F/NL-G-F^·*Mutyh*^−/−^ hippocampus was markedly reduced compared to that in the *App*^NL-G-F/NL-G-F^ hippocampus. These observations were further confirmed by an immunohistochemical analysis of CD68 and qRT-PCR of *Cd68*. Thus, we concluded that MUTYH actively contributes to microglial activation in the early phase of AD pathology, thereby affecting memory impairment.

Some signals generated under AD pathological conditions promote transition from homeostatic microglia to DAM in two steps: stage 1 and stage 2. We found that the expression of several marker genes for both stage 1 and 2 DAM, such as *Ctsd*, *Lyz2*, *Timp2*, *Cd68*, *Cst7*, *Trem2*, and *Ccl6*, was significantly decreased in the *App*^NL-G-F/NL-G-F^·*Mutyh*^−/−^ hippocampus compared to the *App*^NL-G-F/NL-G-F^ hippocampus. Among them, TREM2 is known to be closely associated with AD, and its signaling is required for stage 2 (but not stage 1) DAM induction; TREM2-dependent signaling involves the upregulation of the lysosomal, phagocytic, and lipid metabolism pathways [69, 70]. Furthermore, functional annotation clustering of genes revealed that genes related to lysosomes exhibited the highest enrichment among genes whose expression was significantly reduced in the *App*^NL-G-F/NL-G-F^·*Mutyh*^−/−^ hippocampus compared to the *App*^NL-G-F/NL-G-F^ hippocampus, thus suggesting that the microglia in the former had a much lower phagocytic activity than those in the latter. Taken together, our data indicate that MUTYH is involved in microglial response pathways from the early stage of AD pathology and contributes to phagocytosis of DAM. These observations were further confirmed by the 3D morphological analysis of microglia, especially those surrounding amyloid plaques.

We also found in the present study that the density of BrdU-positive cells in the SGZ and GCL was decreased in six-month-old *App*^NL-G-F/NL-G-F^ female mice compared with wild-type mice, and MUTYH deficiency increased the density of BrdU-positive cells. These results indicate that the MUTYH function is detrimental to hippocampal neurogenesis in the *App*^NL-G-F/NL-G-F^ mice. In a healthy adult hippocampus, homeostatic highly ramified microglia, such as those observed in the six-month-old wild-type brain, are an essential component of the neurogenic niche in the SGZ and GCL for the maintenance of homeostasis [71, 72]. They also secrete a number of growth factors and neurotrophic factors that promote neurogenesis [72–74]. Decreased hippocampal neurogenesis has been reported in several AD model mice [53, 54], as we observed in the *App*^NL-G-F/NL-G-F^ hippocampus, wherein amoeboid-shaped DAM dominated. Minocycline treatment reportedly suppresses microglial activation and improves neurogenesis as well as the cognitive function but not A*β* deposition in APP/PS1 mice [75], similar to the case with MUTYH deficiency in *App*^NL-G-F/NL-G-F^ mice. DAM possess enhanced phagocytic programs for the A*β* uptake, which are considered to be neuroprotective; however, such enhanced phagocytosis may induce aberrant synaptic pruning, leading to neuronal dysfunction. DAM also produce directly neurotoxic substances, such as proinflammatory cytokines and reactive oxygen species, thereby enhancing neuroinflammation [51, 52, 73]. Taken together, our results suggest that MUTYH deficiency suppresses microglial activation and ameliorates the detrimental consequences of DAM, thereby reducing the negative phenotypes associated with neuronal dysfunction and neuroinflammation as well as reduced neurogenesis under AD pathological conditions.

Although the detailed mechanisms regarding how MUTYH accelerates microgliosis and adversely affects neurogenesis need to be clarified, low-molecular-weight compounds inhibiting the MUTYH function or depleting MUTYH protein may be potential candidates for the development of therapeutic agents for preclinical or early-phase AD. Recently, the insulin secretagogues “sulfonylureas,” such as acetohexamide and glimepiride but not gliclazide or glibenclamide, were reported to induce MUTYH degradation dependent on proteasomes [76]; therefore, our hypothesis suggests that the inhibition or depletion of MUTYH in the brain by such drugs can ameliorate the AD pathogenesis which may be confirmed in the near future using these compounds, even with clinical subjects. Moreover, mutations in the *MUTYH* gene are known to predispose patients to the development of both colorectal polyposis and cancer [42, 77]; however, our hypothesis suggests that they may be less susceptible to AD with efficient care for carcinogenesis.

We recently demonstrated that type 2 diabetes mellitus (T2DM) induced by a high-fat diet causes an impaired cognitive function in a mild preclinical AD model of *App*^NL-F/NL-F^ mice accompanied by marked increases in both microgliosis and 8-oxoG accumulation as well as insulin resistance in the hippocampus [78]. The AD brain exhibits insulin resistance, and T2DM is a major risk factor for AD [1–3, 31]; therefore, insulin secretagogues which can induce MUTYH degradation are promising therapeutic agents for the prevention of the AD pathogenesis. Because patients with mutations in the *MUTYH* gene do not predispose to brain tumors [42, 77] and since we observed intestinal tumors but neither brain tumors nor neurodegeneration in MUTYH-deficient mice [25, 33], the brain-specific delivery of drugs should be considered in order to minimize such adverse effects, including hypoglycemia.

## 5. Conclusions

In conclusion, we propose that MUTYH, which is expressed in the hippocampus of AD and non-AD patients, actively contributes to memory impairment by inducing microglial activation with poor neurogenesis and is a novel therapeutic target for early-stage AD, as MUTYH deficiency is highly beneficial for ameliorating AD pathogenesis.

## Figures and Tables

**Table 1:** Expression of multiforms of *MUTYH* mRNA in the human hippocampus with or without AD pathology.

Transcript ID	1^st^ exon	Splicing in exon 3	RT-PCR product (base)^a^	MUTYH protein isoform no.	Non-AD (*n* = 10)		AD (*n* = 8)		LR^c^ test	
					Mean^b^	SD	Mean^b^	SD	*p* value	FDR
NM_012222.3	*α*	1	413	1	0.022	0.065	0.175	0.257	0.096	0.612
NM_001293190.2	*α*	2	383	6	0.067	0.138	0.192	0.245	0.358	1.000
NM_001048171.2	*α*	3	380	2	0.320	0.588	0.312	0.319	0.810	1.000
NM_001293192.2	*α*	4	316	8	0.117	0.188	0.372	0.371	0.145	0.734
NM_001128425.2	*α*	5	422	5	0.155	0.252	0.145	0.104	0.783	1.000
NM_001293191.2	*β*	1	278	7	0.066	0.153	0.098	0.162	0.846	1.000
NM_001048174.2	*β*	3	245	4	0.679	1.152	0.360	0.747	0.581	1.000
NM_001293195.2	*β*	5	395	4	0.126	0.087	0.098	0.114	0.753	1.000
NM_001048172.2	*γ*	2	316	3	0.204	0.466	0.095	0.164	0.569	1.000
NM_001048173.2	*γ*	3	313	4	0.431	0.584	0.267	0.369	0.690	1.000
NM_001293196.2	*γ*	4	249	8	0.093	0.105	0.218	0.290	0.393	1.000
NM_001350650.2	*α*	3	380	9	0.078	0.207	0.062	0.087	0.767	1.000
NM_001350651.2	*α*	4	316	9	0.026	0.055	0.051	0.073	0.709	1.000
NR_146882.2	*α*	5	380	478 aa (52.7 kDa)	0.548	0.385	1.579	0.836	0.070	0.525
NR_146883.2	*γ*	3	313	363 aa (39.6 kDa)	0.372	0.669	0.308	0.870	0.860	1.000

MSTRG.709.1	*α*	3	380	349 aa (37.8 kDa)	0.343	0.530	0.677	0.631	0.369	1.000
MSTRG.709.2	*γ*	3	313	349 aa (37.8 kDa)	0.869	1.181	0.511	0.741	0.761	1.000
MSTRG.709.3	Novel 1^st^ exon	4	NA	267 aa (29.4 kDa)	1.067	0.865	1.188	0.656	0.498	1.000
MSTRG.709.4	*α*′	3	396	349 aa (37.8 kDa)	1.175	0.806	1.123	0.731	0.787	1.000
MSTRG.709.20	*α*	4	316	NC	0.673	1.035	0.645	0.483	0.835	1.000
MSTRG.709.21	Exon 3	1	NA	NC	0.611	0.717	1.379	1.483	0.179	0.806

Expression level of *MUTYH* at the gene level					11.684	4.594	13.615	7.139	**0.005**	0.103

^a^Sizes of RT-PCR products expected in Supplementary Figure S2 are shown. ^b^Expression levels are shown by transcripts per million. ^c^Likelihood ratio test using the R package edgeR; bold indicates *p* values < 0.05. RT-PCR = reverse transcription PCR; AD = Alzheimer's disease; SD = standard deviation; FDA = false discovery rate; NA = not amplified; NC = noncoding.

**Table 2:** The altered expression of marker genes for various brain cell types in the hippocampi of 6-month-old female wild-type, *App*^NL-G-F/NL-G-F^, and *App*^NL-G-F/NL-G-F^·*Mutyh*^−/−^ mice.

Cell type	Marker gene	Relative expression (% wild type)	
		*App* ^NL-G-F/NL-G-F^	*App* ^NL-G-F/NL-G-F^·*Mutyh*^−/−^
NSCs/NPCs	*Ascl1*	87.06	*77.38*
	*Bmp4*	87.66	99.31
	*Dcx*	95.93	97.27
	*Egf*	106.44	106.44
	*Igf2*	144.39	125.7
	*Notch2*	104.97	101.4
	*Shh*	93.3	90.75
	*Sox2*	100	90.75
	Mean	102.47	98.63
	SD	17.21	13.13

Neurons	*Chga*	104.25	*92.02*
	*Eno2*	103.53	102.81
	*Nefh*	100	106.44
	*Nefl*	103.53	108.67
	*Nefm*	104.25	107.18
	*Rbfox3*	93.3	109.43
	*Snap25*	98.62	100
	*Syp*	96.59	95.93
	*Syt1*	91.38	92.02
	*Tubb2a*	97.94	93.95
	Mean	101.64	100.87
	SD	4.4	6.62

Astrocytes	*Gfap*	*141.42*	*155.83*
	*S100b*	102.81	98.62
	*Glul*	97.27	98.62
	*Slc1a2*	92.02	100
	*Slc1a3*	99.31	101.4
	*Aqp4*	99.31	99.31
	Mean	105.36	108.96
	SD	16.45	20.98

Oligodendrocytes	*Mbp*	96.59	90.13
	*Sox10*	91.38	86.45
	*Mog*	**93.3**	***80.11***
	*Cnp*	94.61	92.02
	*Apc*	101.4	101.4
	*Gstp1*	**102.81**	**90.75**
	*Plp1*	96.59	95.93
	*Mag*	**97.94**	***87.06***
	Mean	96.83	90.48
	SD	3.62	5.99

Microglia	*Aif1*	*110.96*	*113.29*
	*Cd68*	***197.25***	***171.71***
	*Lgals3*	**106.44**	**91.38**
	*Adgre1*	*122.26*	*123.11*
	Mean	134.23	124.87
	SD	36.84	29.38

Genes whose expression differed significantly between *App*^NL-G-F/NL-G-F^ and *App*^NL-G-F/NL-G-F^·*Mutyh*^−/−^ are shown in bold, and genes that differed significantly from wild-type samples are italics or bold-italics. NSC = neural stem cell; NPC = neural progenitor cell; SD = standard deviation.

**Table 3:** The altered expression of microglial stage-specific genes in the hippocampi of 6-month-old female wild-type, *App*^NL-G-F/NL-G-F^, and *App*^NL-G-F/NL-G-F^·*Mutyh*^−/−^ mice.

Gene	Average expression (log2)			*F*-test	*App* ^NL-G-F/NL-G-F^ vs. wild type		*App* ^NL-G-F/NL-G-F^ ·*Mutyh*^−/−^ vs. *App*^NL-G-F/NL-G-F^		*App* ^NL-G-F/NL-G-F^ ·*Mutyh*^−/−^ vs. wild type		Cell
Symbol^b^	Wild type	*App* ^NL-G-F/NL-G-F^	*App* ^NL-G-F/NL-G-F^·*Mutyh*^−/−^	*p* value^a^	Fold change	*p* value^a^	Fold change	*p* value^a^	Fold change	*p* value^a^	Type
*Hexb*	9.55	10.14	10.17	3.30**E** − 05	1.50	2.39**E** − 05	1.02	0.7434	1.53	3.33**E** − 05	Homeostatic
*C1qb*	9.33	9.87	10.01	4.84**E** − 05	1.45	6.45**E** − 05	1.10	0.4371	1.60	2.81**E** − 05	
*Ctss*	9.62	10.33	10.48	8.50**E** − 05	1.65	**0.0001**	1.11	0.4902	1.82	4.99**E** − 05	
*C1qc*	7.08	7.72	7.62	**0.0004**	1.56	**0.0002**	-1.07	0.1922	1.46	**0.001**	
*Csf1r*	8.66	8.97	9.03	**0.0006**	1.24	**0.0006**	1.04	0.7342	1.29	**0.0004**	
*Olfml3*	7.13	7.50	7.38	**0.003**	1.29	**0.001**	-1.08	0.1532	1.19	**0.0101**	
*C1qa*	7.99	8.66	8.91	**0.0042**	1.60	**0.0034**	1.18	0.9048	1.89	**0.0028**	
***Sparc***	9.57	9.77	9.46	**0.0422**	1.15	0.0919	-1.24	**0.015**	-1.08	0.2911	
*Tmsb4x*	7.91	8.00	8.07	0.0917	1.06	0.1379	1.05	0.4195	1.11	**0.0357**	
*Cx3cr1*	8.22	8.38	8.31	0.1034	1.12	**0.0383**	-1.05	0.2926	1.06	0.2214	
*P2ry12*	7.92	8.00	8.09	0.163	1.06	0.8204	1.06	0.085	1.12	0.1228	
*Cst3*	11.42	11.51	11.36	0.3314	1.06	**0.1883**	-1.11	0.2231	-1.05	0.9107	

*Tyrobp*	6.61	7.37	7.17	8.50**E** − 05	1.69	6.45**E** − 05	-1.14	0.1519	1.47	8.60**E** − 05	Stage 1 DAM
***Ctsd***	10.90	11.60	11.31	**0.0003**	1.63	6.45**E** − 05	-1.23	**0.0034**	1.33	**0.0191**	
***Lyz2***	5.96	7.10	6.67	**0.0013**	2.21	**0.0004**	-1.35	**0.0314**	1.64	**0.0142**	
*B2m*	11.24	11.57	11.68	**0.0335**	1.26	**0.0475**	1.08	0.4576	1.36	**0.0136**	
*Ctsb*	11.65	11.76	11.81	0.0677	1.07	0.053	1.04	0.8195	1.11	**0.0362**	
***Timp2***	10.13	10.11	9.25	0.0753	-1.01	0.7337	-1.82	**0.0371**	-1.84	0.0655	
*Cstb*	7.43	7.57	7.34	0.1574	1.10	0.2916	-1.18	0.0614	-1.07	0.3341	
*H2-D1*	5.54	5.71	5.85	0.3992	1.13	0.1978	1.11	0.6835	1.24	0.357	
*Apoe*	7.82	7.89	7.81	0.5273	1.05	0.4561	-1.06	0.2807	-1.01	0.7201	
*Fth1*	13.00	13.03	13.03	0.7389	1.02	0.4795	1.00	0.9052	1.02	0.5536	

***Cd68***	7.90	8.88	8.68	3.18**E** − 07	1.97	1.21**E** − 07	-1.15	**0.0048**	1.71	1.25**E** − 06	Stage 2 DAM
*Clec7a*	4.53	6.36	6.52	6.46**E** − 07	3.56	8.43**E** − 07	1.11	0.3111	3.97	4.17**E** − 07	
***Cst7***	5.53	7.64	6.95	2.62**E** − 06	4.33	9.18**E** − 07	-1.61	**0.0089**	2.69	1.27**E** − 05	
***Trem2***	7.01	7.73	7.50	4.52**E** − 06	1.65	1.73**E** − 06	-1.17	**0.0246**	1.41	1.61**E** − 05	
*Itgax*	5.13	5.59	5.79	6.78**E** − 05	1.37	**0.0002**	1.15	0.0709	1.58	2.52**E** − 05	
***Ccl6***	4.78	5.90	5.46	**0.0003**	2.18	9.03**E** − 05	-1.36	**0.0312**	1.61	**0.0023**	
*Cd52*	5.77	6.36	6.33	**0.0017**	1.51	**0.0007**	-1.02	0.2591	1.48	**0.0037**	
*Gusb*	7.07	7.34	7.36	**0.0087**	1.21	**0.0049**	1.01	0.7505	1.22	**0.0081**	
*Ctsz*	9.08	9.55	9.39	**0.0108**	1.38	**0.0037**	-1.12	0.2169	1.24	**0.0301**	
*Cd9*	8.59	8.96	8.84	**0.0441**	1.29	**0.0293**	-1.08	0.982	1.19	**0.0282**	
*Cd63*	8.48	8.90	8.61	0.0745	1.34	**0.0289**	-1.22	0.4213	1.09	0.1122	
*Ctsa*	9.29	9.41	9.37	0.1195	1.09	0.0615	-1.03	0.7992	1.06	0.0935	
*Serpine2*	9.45	9.59	9.47	0.1448	1.10	0.056	-1.08	0.3433	1.02	0.2617	
*Cadm1*	9.57	9.44	9.11	0.1544	-1.09	0.8814	-1.26	0.1083	-1.38	0.085	
*Csf1*	7.39	7.53	7.57	0.2612	1.10	0.1746	1.03	0.9153	1.13	0.1479	
*Axl*	8.11	8.19	8.17	0.2701	1.06	0.1175	-1.02	0.5009	1.04	0.3287	
*Ctsl*	8.47	8.62	8.50	0.2709	1.11	0.1201	-1.09	0.2994	1.02	0.5517	
*Spp1*	5.99	5.97	5.99	0.294	-1.01	0.1361	1.02	0.6252	1.00	0.2865	
*Lpl*	7.29	7.33	7.25	0.3032	1.03	0.3701	-1.05	0.1342	-1.02	0.4987	
*Ank*	9.54	9.55	9.61	0.7864	1.00	0.9266	1.04	0.5923	1.04	0.5321	
*Hif1a*	8.67	8.69	8.69	0.8123	1.01	0.5354	1.00	0.8183	1.01	0.6928	

^a^ *p* values < 0.05 are indicated in bold. ^b^Genes whose expression differed significantly altered between *App*^NL-G-F/NL-G-F^ and *App*^NL-G-F/NL-G-F^·*Mutyh*^−/−^ are shown in bold. DAM=disease-associated microglia.

**Table 4:** Functional annotation clustering of genes whose expression was significantly altered by MUTYH deficiency in the *App*^NL-G-F/NL-G-F^ hippocampus.

Category	Term	Number of genes^a^	% of genes	Fold enrichment	*p* value^b^	Genes	Up^c^	Down^d^
Annotation cluster 1 (enrichment score: 2.16)								
UP_KEYWORDS	Lysosome	6	5.83	5.58	4.35*E* − 03	***Npc2***, ***Psap***, ***Cd68***, ***Ctsd***, ***Rnaset2b***, ***Rnaset2a***	0	6
GOTERM_CC_DIRECT	GO:0005764 lysosome	7	6.80	4.47	4.61*E* − 03	***Npc2***, ***Psap***, ***Gpc3***, ***Cd68***, ***Ctsd***, ***Rnaset2b***, ***Rnaset2a***	0	7
KEGG_PATHWAY	mmu04142 lysosome	4	3.88	7.20	1.63*E* − 02	***Npc2***, ***Psap***, ***Cd68***, ***Ctsd***	0	4

Annotation cluster 2 (enrichment score: 1.71)								
UP_KEYWORDS	Secreted	17	16.50	2.33	2.03*E* − 03	***Lyz2***, ***Trem2***, *Serpina3n*, ***Cst7***, ***Spata6***, ***Rnaset2b***, ***Tnfsf13b***, ***Rnaset2a***, ***Ccl6***, ***Npc2***, *Arsj*, ***Smoc1***, ***Psap***, ***Gpc3***, ***Ctsd***, ***Gpha2***, *Havcr2*	3	14
UP_KEYWORDS	Glycoprotein	29	28.16	1.76	2.15*E* − 03	***Slc22a4***, ***Ly6c2***, ***Itgb5***, *Kcna4*, ***Il6ra***, *Gpr83*, ***Trem2***, ***Cst7***, ***Rnaset2b***, ***Rnaset2a***, ***Tnfsf13b***, *Foxred2*, *Grm2*, ***Tmem145***, *Arsj*, ***Psap***, ***Gpc3***, *Chsy3*, ***Gpha2***, ***Ctsd***, ***Prom2***, *Havcr2*, *Entpd1*, *Serpina3n*, ***Krt18***, ***Npc2***, ***Smoc1***, ***Plxnb2***, ***Cd68***	9	20
GOTERM_CC_DIRECT	GO:0005576 extracellular region	16	15.53	1.93	1.58*E* − 02	***Lyz2***, ***Il6ra***, ***Trem2***, *Serpina3n*, ***Cst7***, ***Spata6***, ***Tnfsf13b***, ***Ccl6***, ***Npc2***, *Arsj*, ***Smoc1***, ***Psap***, ***Gpc3***, ***Ctsd***, ***Gpha2***, *Havcr2*	3	13
UP_KEYWORDS	Disulfide bond	22	21.36	1.63	2.21*E* − 02	***Ly6c2***, *Entpd1*, ***Itgb5***, ***Lyz2***, ***Il6ra***, ***Trem2***, *Gpr83*, ***Cst7***, ***Rnaset2b***, ***Tnfsf13b***, ***Rnaset2a***, *Grm2*, ***Ccl6***, ***Npc2***, ***Smoc1***, ***Psap***, ***Cdk2ap1***, ***Plxnb2***, ***Cd68***, ***Ctsd***, ***Gpha2***, *Havcr2*	4	18
UP_SEQ_FEATURE	Signal peptide	24	23.30	1.52	3.16*E* − 02	***Ly6c2***, ***Itgb5***, ***Lyz2***, ***Il6ra***, ***Trem2***, *Gpr83*, *Serpina3n*, ***Cst7***, ***Spata6***, ***Rnaset2b***, ***Rnaset2a***, *Foxred2*, *Grm2*, ***Ccl6***, ***Npc2***, *Arsj*, ***Smoc1***, ***Psap***, ***Gpc3***, ***Cd68***, ***Ctsd***, ***Gpha2***, ***Prom2***, *Havcr2*	6	18
UP_KEYWORDS	Signal	28	27.18	1.43	4.00*E* − 02	***Ly6c2***, ***Itgb5***, ***Lyz2***, ***Il6ra***, *Gpr83*, ***Trem2***, ***Cst7***, ***Spata6***, ***Rnaset2b***, ***Rnaset2a***, *Foxred2*, *Grm2*, ***Tmem145***, ***Ccl6***, *Arsj*, ***Psap***, ***Gpc3***, ***Gpha2***, ***Ctsd***, ***Prom2***, *Havcr2*, *Serpina3n*, ***Npc2***, ***Smoc1***, *Pcdhb6*, ***Plxnb2***, ***Mxra7***, ***Cd68***	7	21
UP_SEQ_FEATURE	Glycosylation site: N-linked (GlcNAc…)	26	25.24	1.44	4.19*E* − 02	***Slc22a4***, ***Itgb5***, *Kcna4*, ***Il6ra***, *Gpr83*, ***Trem2***, ***Cst7***, ***Rnaset2b***, ***Rnaset2a***, ***Tnfsf13b***, *Foxred2*, *Grm2*, ***Tmem145***, *Arsj*, ***Psap***, ***Gpc3***, *Chsy3*, ***Gpha2***, ***Ctsd***, ***Prom2***, *Havcr2*, *Entpd1*, *Serpina3n*, ***Npc2***, ***Smoc1***, ***Cd68***	9	17
GOTERM_CC_DIRECT	GO:0005615 extracellular space	13	12.62	1.83	4.75*E* − 02	*Entpd1*, ***Lyz2***, ***Il6ra***, *Serpina3n*, *Akr1b3*, ***Cst7***, ***Rnaset2b***, ***Tnfsf13b***, ***Rnaset2a***, ***Ccl6***, ***Psap***, ***Gpc3***, ***Ctsd***	3	10

Annotation cluster 3 (enrichment score: 1.54)								
INTERPRO	IPR011992 EF-hand-like domain	6	5.83	4.76	8.31*E* − 03	***Spef2***, ***Cracr2b***, ***Tnnc1***, ***Smoc1***, ***Lpcat2***, ***Slc25a13***	0	6
UP_KEYWORDS	Calcium	10	9.71	2.80	8.95*E* − 03	*Entpd1*, *Pcdhb2*, *Tpm4*, ***Anxa3***, ***Tnnc1***, *Arsj*, ***Smoc1***, ***Lpcat2***, *Pcdhb6*, ***Slc25a13***	5	5
UP_SEQ_FEATURE	Domain: EF-hand 2	5	4.85	5.72	1.11*E* − 02	***Cracr2b***, ***Tnnc1***, ***Smoc1***, ***Lpcat2***, ***Slc25a13***	0	5
UP_SEQ_FEATURE	Domain: EF-hand 1	5	4.85	5.69	1.13*E* − 02	***Cracr2b***, ***Tnnc1***, ***Smoc1***, ***Lpcat2***, ***Slc25a13***	0	5
UP_SEQ_FEATURE	Calcium-binding region: 2	4	3.88	6.95	1.95*E* − 02	***Tnnc1***, ***Smoc1***, ***Lpcat2***, ***Slc25a13***	0	4
UP_SEQ_FEATURE	Calcium-binding region: 1	4	3.88	6.28	2.53*E* − 02	***Tnnc1***, ***Smoc1***, ***Lpcat2***, ***Slc25a13***	0	4
GOTERM_MF_DIRECT	GO:0005509 calcium ion binding	8	7.77	2.56	3.44*E* − 02	***Cracr2b***, *Pcdhb2*, ***Anxa3***, ***Tnnc1***, ***Smoc1***, ***Lpcat2***, *Pcdhb6*, ***Slc25a13***	2	6

^a^Among all transcript clusters (34,472), 1,164 clusters with row expression intensity > 50 exhibit significantly different expression levels among wild-type, *App*^NL-G-F/NL-G-F^, and *App*^NL-G-F/NL-G-F^·*Mutyh*^−/−^ hippocampi (ANOVA with eBayes correction, *F*-test *p* < 0.05). Among these clusters, 138 exhibit significant difference between both wild-type vs. *App*^NL-G-F/NL-G-F^ and *App*^NL-G-F/NL-G-F^ vs. *App*^NL-G-F/NL-G-F^·*Mutyh*^−/−^ samples. Out of 138 clusters, 103 genes were functionally annotated and clustered in DAVID with the default classification stringency (medium). Top 3 clusters with enrichment score > 1.3 are shown. ^b^The enrichment *p* value (EASE score) of each annotation term in a given group with modified Fisher's exact test. Terms with *p* < 0.05 are shown. ^c^Number of genes whose expression levels in *App*^NL-G-F/NL-G-F^·*Mutyh*^−/−^ are significantly increased compared to *App*^NL-G-F/NL-G-F^ samples. ^d^Number of genes whose expression levels in *App*^NL-G-F/NL-G-F^·*Mutyh*^−/−^are significantly decreased compared to *App*^NL-G-F/NL-G-F^ samples.

## Data Availability

Illumina high-throughput RNA sequencing data were deposited in the GEO database (accession number GSE173955), and all microarray data were deposited in the GEO database (accession numbers GSE157161 and GSE157766). All other data are available upon request.

## Supplementary Materials

Supplementary Materials Figure S1: immunohistochemistry without a primary antibody as a negative control. Figure S2: multiforms of *MUTYH* mRNA detected in the human brain. Figure S3: spontaneous locomotor activity of wild-type, *App*^NL-G-F/NL-G-F^, and *App*^NL-G-F/NL-G-F^·*Mutyh*^−/−^ mice. Figure S4: open-field test in wild-type, *App*^NL-G-F/NL-G-F^, and *App*^NL-G-F/NL-G-F^·*Mutyh*^−/−^ mice. Figure S5: the item discrimination index during the novel object recognition test of wild-type, *App*^NL-G-F/NL-G-F^, and *App*^NL-G-F/NL-G-F^·*Mutyh*^−/−^ mice. Figure S6: Western blot analyses of SDS-soluble A*β* peptide in six-month-old female mouse hippocampal extracts. Figure S7: immunofluorescence microscopy in the hippocampus from six-month-old female *App*^NL-G-F/NL-G-F^ mice. Table S1: list of human autopsy brain samples. Table S2: expression of multiforms of *MUTYH* mRNA in the human hippocampus with or without AD pathology. Table S3: the altered expression of marker genes for three types of astrocytes in the hippocampi of six-month-old female wild-type, *App*^NL-G-F/NL-G-F^, and *App*^NL-G-F/NL-G-F^·*Mutyh*^−/−^ mice. Table S4: list of 103 genes subjected to functional annotation clustering by DAVID.
