# Corrigendum to “MUTYH Actively Contributes to Microglial Activation and Impaired Neurogenesis in the Pathogenesis of Alzheimer's Disease”

**DOI:** 10.1155/2023/9794081

**Published:** 2023-02-06

**Authors:** Yuri Mizuno, Nona Abolhassani, Guianfranco Mazzei, Kunihiko Sakumi, Takashi Saito, Takaomi C. Saido, Toshiharu Ninomiya, Toru Iwaki, Ryo Yamasaki, Jun-ichi Kira, Yusaku Nakabeppu

**Affiliations:** ^1^Division of Neurofunctional Genomics, Department of Immunobiology and Neuroscience, Medical Institute of Bioregulation, Kyushu University, Fukuoka 812-8582, Japan; ^2^Department of Neurology, Neurological Institute, Graduate School of Medical Sciences, Kyushu University, Fukuoka 812-8582, Japan; ^3^Department of Neurocognitive Science, Institute of Brain Science, Nagoya City University Graduate School of Medical Science, Aichi 467-8601, Japan; ^4^Laboratory for Proteolytic Neuroscience, RIKEN Center for Brain Science, Saitama 351-0198, Japan; ^5^Department of Epidemiology and Public Health, Graduate School of Medical Sciences, Kyushu University, Fukuoka 812-8582, Japan; ^6^Department of Neuropathology, Graduate School of Medical Sciences, Kyushu University, Fukuoka 812-8582, Japan; ^7^Translational Neuroscience Center, Graduate School of Medicine and School of Pharmacy at Fukuoka, International University of Health and Welfare, Fukuoka 831-8501, Japan

In the article titled “MUTYH Actively Contributes to Microglial Activation and Impaired Neurogenesis in the Pathogenesis of Alzheimer's Disease,” [1] the authors identified an error in the legend of Figure 8. The article should therefore be corrected as follows: “Scale bar = 100 *μ*m” should be corrected to “Scale bar = 20 *μ*m.”

The corrected legend is shown in the following:

Figure 8: MUTYH deficiency suppressed the morphological alteration of microglia in the *App*^NL-G-F/NL-G-F^ brain. (a) Immunofluorescent micrograph of hippocampal microglia stained for Iba-1 (green) in six-month-old female wild-type (Wt), *App*^NL-G-F/NL-G-F^ (NL-G-F), and *App*^NL-G-F/NL-G-F^·*Mutyh^−/−^* (NL-G-F·Mutyh) mice. Nuclei were counterstained with DAPI (blue). Scale bar = 20 *μ*m. (b) Three-dimensional reconstruction of microglia surrounding A*β* plaque in the six-month-old female hippocampus. Scale bar = 20 *μ*m.
