# Supplementary material for: MUTYH Actively Contributes to Microglial Activation and Impaired Neurogenesis in the Pathogenesis of Alzheimer's Disease
Source: Oxid Med Cell Longev. 2021 Dec 21;2021:8635088. doi: 10.1155/2021/8635088 (PMC8714343; doi:10.1155/2021/8635088)
Supplement: Supplementary Materials — Figure S1: immunohistochemistry without a primary antibody as a negative control. Figure S2: multiforms of MUTYH mRNA detected in the human brain. Figure S3: spontaneous locomotor activity of wild-type, AppNL-G-F/NL-G-F, and AppNL-G-F/NL-G-F·Mutyh−/− mice. Figure S4: open-field test in wild-type, AppNL-G-F/NL-G-F, and AppNL-G-F/NL-G-F·Mutyh−/− mice. Figure S5: the item discrimination index during the novel object recognition test of wild-type, AppNL-G-F/NL-G-F, and AppNL-G-F/NL-G-F·Mutyh−/− mice. Figure S6: Western blot analyses of SDS-soluble Aβ peptide in six-month-old female mouse hippocampal extracts. Figure S7: immunofluorescence microscopy in the hippocampus from six-month-old female AppNL-G-F/NL-G-F mice. Table S1: list of human autopsy brain samples. Table S2: expression of multiforms of MUTYH mRNA in the human hippocampus with or without AD pathology. Table S3: the altered expression of marker genes for three types of astrocytes in the hippocampi of six-month-old female wild-type, AppNL-G-F/NL-G-F, and AppNL-G-F/NL-G-F·Mutyh−/− mice. Table S4: list of 103 genes subjected to functional annotation clustering by DAVID. [file 8635088.f1.zip › Mizuno_OMCL_SM.pdf]

Supplementary Materials

MUTYH actively contributes to microglial activation and impaired neurogenesis in the pathogenesis of Alzheimer's disease

Yuri Mizuno,[1,2,#] Nona Abolhassani,[1,#] Guianfranco Mazzei,[1] Kunihiko Sakumi,[1] Takashi Saito,[3,4] Takaomi C. Saido,[4] Toshiharu Ninomiya,[5] Toru Iwaki,[6] Ryo Yamasaki,[2] Jun-ichi Kira,[2,7] Yusaku Nakabeppu[1]

[#]Yuri Mizuno and Nona Abolhassani contributed equally to this work.

[1] Division of Neurofunctional Genomics, Department of Immunobiology and Neuroscience, Medical Institute of Bioregulation, Kyushu University, Fukuoka 812-8582, Japan.
[2] Department of Neurology, Neurological Institute, Graduate School of Medical Sciences, Kyushu University, Fukuoka 812-8582, Japan.
[3] Department of Neurocognitive Science, Institute of Brain Science, Nagoya City University Graduate School of Medical Science, Aichi 467-8601, Japan.
[4] Laboratory for Proteolytic Neuroscience, RIKEN Center for Brain Science, Saitama 351-0198, Japan.
[5] Department of Epidemiology and Public Health, Graduate School of Medical Sciences, Kyushu University, Fukuoka 812-8582, Japan.
[6] Department of Neuropathology, Graduate School of Medical Sciences, Kyushu University, Fukuoka 812-8582, Japan.
[7] Translational Neuroscience Center, Graduate School of Medicine, and School of Pharmacy at Fukuoka, International University of Health and Welfare, Fukuoka 831-8501, Japan.

Correspondence should be addressed to Yusaku Nakabeppu; yusaku@bioreg.kyushu-u.ac.jp

This file contains Supplementary Materials and Methods, References, and Supplementary Figures (Fig. S1 to Fig. S7).

Supplementary Materials and Methods

Reverse transcription polymerase chain reaction (RT-PCR) of post-mortem human brain RNA samples

Hippocampal RNA samples that had been prepared previously were reverse-transcribed to first-strand cDNA [1]. The cDNAs were amplified using a previously designed common downstream primer (*MUTYH*3′-4: 5′-AGGCTGTGACTTCAGCTAC-3′) and an upstream primer specific for each type of *MUTYH* mRNA (type α-5′: 5′-GAGGAGCCTCTAGAACTATGA-3′, type β-5′: 5′-CTCCGTGTTCTGCTGTCTTC-3′, type γ-5′: 5′-CGCTAATTGCCTATTGGCCTGT-3′) [2, 3]. PCR was performed in a reaction mixture containing 1 μl cDNA, 0.1 U of MightyAmp DNA polymerase (R071B; Takara Bio Inc., Shiga, Japan), 1 μl of each primer, 10 μl of 2×MightyAmp Buffer Ver.2 (R071B; Takara Bio Inc.) and 7 μl of ultrapure water. The initial denaturation was performed at 95°C for 1 min, and then amplification was performed with 35 cycles of denaturation at 95°C for 20 sec, annealing at 57°C for 20 sec, extension at 72°C for 40 sec and additional extension at 72°C for 5 min. PCR products were analyzed by 6% polyacrylamide gel electrophoresis.

Spontaneous locomotor activity

Mice were individually housed for at least 1 week before monitoring spontaneous locomotor activity in the home cage (27×14×15 cm). The locomotor activity was measured as a count of infrared-beam break using an infrared beam sensor (NS-AS01; Neuroscience Inc., Tokyo, Japan) for three days, and data were analyzed with the DAS-008 software program, as described previously [4, 5].

Open-field test

The open-field test was carried out in a square arena (50×50×40 cm, white, non-transparent). The trajectory and behavior of animals for five minutes were recorded and digitized with a video tracking system (Limelight video tracking system; Actimetrics Inc., Wilmette, IL, USA), as described previously [4, 5]. The mice were placed at the center of the apparatus and allowed to explore the open field for five minutes. The apparatus was cleaned with an ethanol wipe after each test. The following variables were measured: total traveling distance, time spent in the center and peripheral zones and the amount of supported rearing in the testing period.

Novel object recognition test

The novel object recognition test was performed as described previously [6]. The open field

box was placed in a dark room illuminated by a dim light (50 lx). The day before the test (Day 0), each mouse was habituated in the apparatus for 5 min. The next day (Day 1), the mouse was allowed to explore two identical objects (animal mascot) placed in the arena for 10-30 min as a sample object exposure. Each object was placed in a symmetrical position within the box, at the same distance (16 cm) from the side wall, and separated by 20 cm. For novel object recognition, 24 h after the sample object exposure, the mouse was exposed to 2 different objects: 1 of the sample objects as a familiar object and 1 novel object, placed within the arena, for 10 min. The time spent exploring each object was determined and calculated as the preference index: (periods contacting novel object) / (periods contacting novel object + periods contacting familiar object).

Western blot analyses of SDS-soluble Aβ in mouse brain samples

Frozen hippocampus samples from 5 *App*$^{NL\text{-}G\text{-}F/NL\text{-}G\text{-}F}$, 5 *App*$^{NL\text{-}G\text{-}F/NL\text{-}G\text{-}F}$•*Mutyh*$^{-/-}$ and 3 wild-type (negative control) six-month-old female mice were sonicated using an ultrasonic processor (XL2020; Qsonica, Newtown, CT, USA) in 2×SDS sample buffer (130 mM Tris-HCl pH 6.8, 4% SDS, 10% glycerol, 4% 2-mercaptoethanol, and 0.01% bromophenol blue) for 30 sec and then centrifuged at 14,500 *g* for 10 min (KITMAN-18; TOMY, Tokyo, Japan). The supernatant fractions were saved as SDS-soluble fractions, and protein concentrations were determined using an XL-Bradford (SDS-PAGE) reagent (KY-1031; Aproscience, Tokushima, Japan). Denatured protein samples (20 µg total protein/lane) were subjected to 10% SDS-PAGE and transferred onto a 0.2-µm nitrocellulose membrane (10600001; Cytiva, Tokyo, Japan). After boiling the membrane in 1×phosphate-buffered saline (PBS) at 95°C for 5 min, the membrane was incubated for 1 h at room temperature in TBST containing 5% nonfat dried milk and then in TBST with 1% nonfat dried milk containing mouse anti-β-Amyloid, 1-16 antibody (1:1000, 803002; Biolegend, Inc., San Diego, CA, USA) overnight at 4°C with gentle shaking. The membrane was then washed and incubated further in TBST with 1% nonfat dried milk containing anti–mouse IgG HRP- linked goat antibody (1:4000, 7076; Cell Signaling Technology, Inc., Danvers, MA, USA) for 1 h at room temperature. Next, bound HRP-linked antibodies on the blots were detected by the chemiluminescence method with EzWestLumi plus (WSE-7120; ATTO, Tokyo, Japan). Digitized images were obtained with an AE-9300 Ez-CaptureMG (ATTO) and analyzed by the densitograph software program CS Analyzer 3 (ATTO). To obtain a loading control, the membrane was stripped and reprobed with mouse anti-β-Actin (1:5000 A5316-2ML; Sigma-Aldrich, Tokyo, Japan) and HRP-linked protein A (1:10000, NA9120-1ML; Cytiva).

References

[1] M. Hokama, S. Oka, J. Leon et al. “Altered expression of diabetes-related genes in Alzheimer's disease brains: the Hisayama study,” *Cerebral Cortex,* vol. 24, no. 9, pp. 2476–2488, 2014.

[2] T. Arai, J. Fukae, T. Hatano et al., “Up-regulation of hMUTYH, a DNA repair enzyme, in the mitochondria of substantia nigra in Parkinson's disease,” *Acta Neuropathologica,* vol. 112, no. 2, pp. 139–145, 2006.

[3] T. Ohtsubo, K. Nishioka, Y. Imaiso et al., “Identification of human MutY homolog (hMYH) as a repair enzyme for 2-hydroxyadenine in DNA and detection of multiple forms of hMYH located in nuclei and mitochondria,” *Nucleic Acids Research*, vol. 28, no. 6, pp. 1355–1364, 2000.

[4] N. Haruyama, K. Sakumi, A. Katogi et al., “8-Oxoguanine accumulation in aged female brain impairs neurogenesis in the dentate gyrus and major island of Calleja, causing sexually dimorphic phenotypes,” *Progress in Neurobiology,* vol. 180, pp. 101613, 2019.

[5] Z. Sheng, S. Oka, D. Tsuchimoto et al., “8-Oxoguanine causes neurodegeneration during MUTYH-mediated DNA base excision repair,” *The Journal of Clinical Investigation*, vol. 122, no. 12, pp. 4344–4361, 2012.

[6] R.A. Bevins and J. Besheer, “Object recognition in rats and mice: a one-trial non-matching-to-sample learning task to study 'recognition memory',” *Nature Protocols,* vol. 1, no. 3, pp. 1306–1311, 2006.

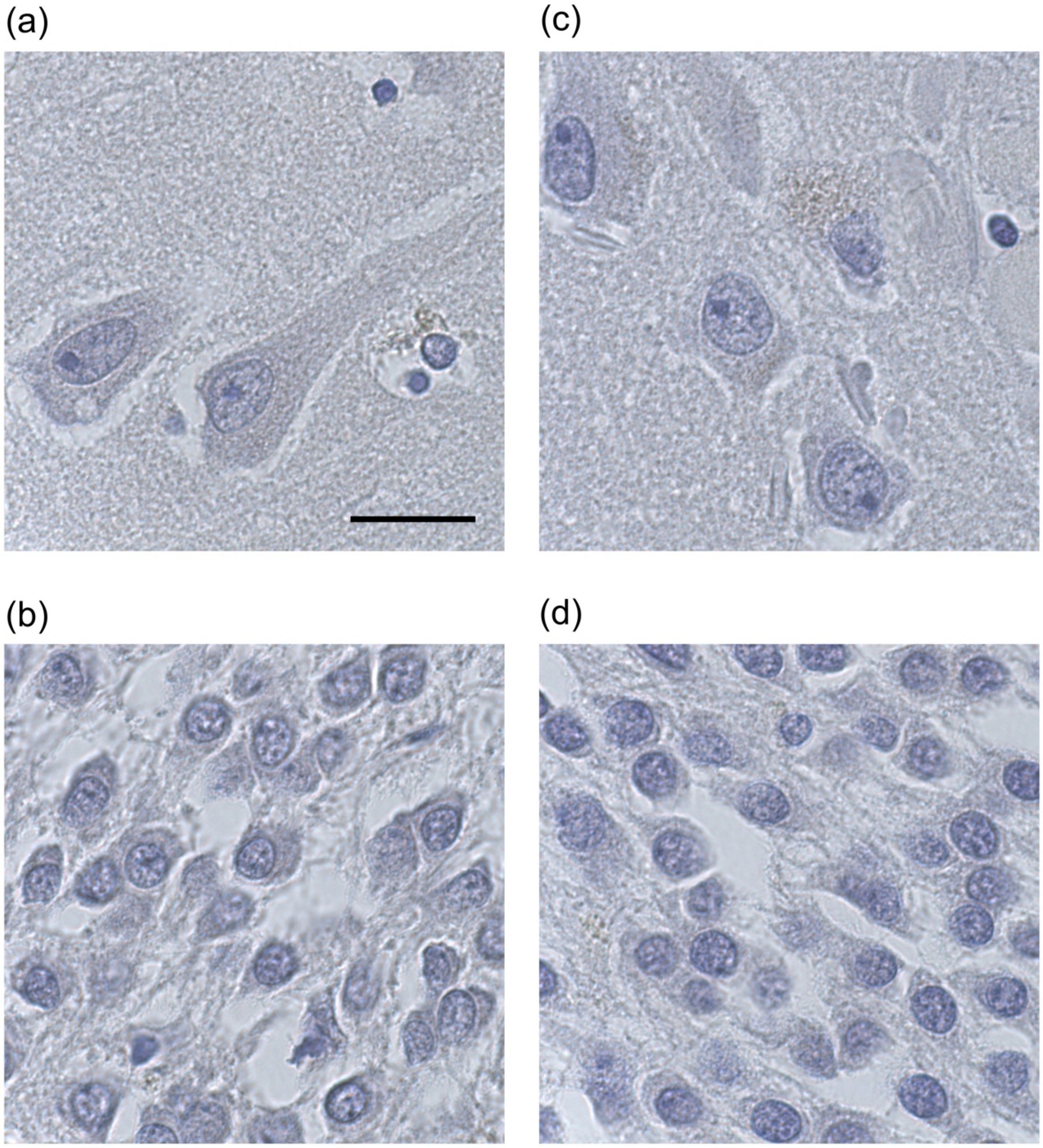

Supplementary Fig. S1. Immunohistochemistry without primary antibody as a negative control. (a), (b) Non-AD brain. (c), (d) AD brain. (a), (c) Pyramidal cell layer in hippocampus CA1 field. (b), (d) Granule cell layer in the dentate gyrus. Nuclei were counter-stained by hematoxylin. Scale bar = 20 μm.

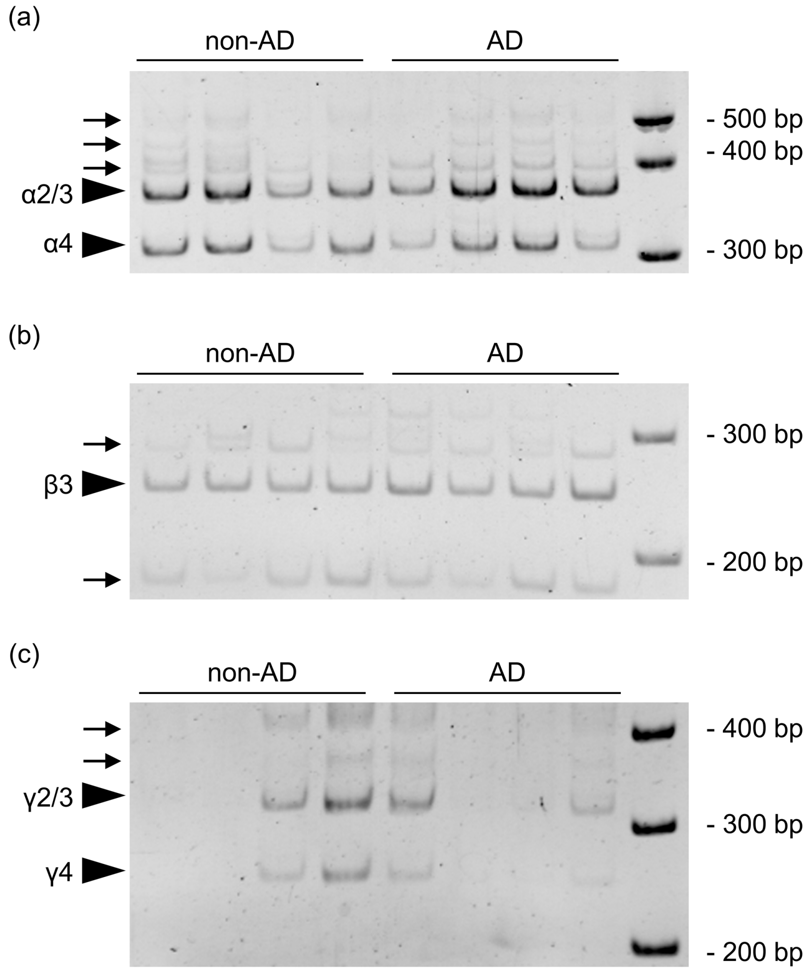

Supplementary Fig. S2. Multiforms of *MUTYH* mRNA detected in human brain. (a)-(c) An RT-PCR analysis. (a) Two major bands were amplified from both non-AD and AD samples with the set of primers for type α mRNAs (arrowheads). Those seemed likely to be type α2 mRNA (383 bp) or α3 mRNA (380 bp) and α4 mRNA (316 bp). (b) Using the set of primers for type β mRNAs, one major band corresponding to type β3 mRNA (245 bp) was amplified from all samples (arrowhead). (c) For type γ mRNAs, two bands corresponding to type γ2 mRNA (316 bp) or γ3 mRNA (313 bp) and γ4 mRNA (249 bp) were amplified from two of the four non-AD samples and two of the four AD samples (arrowheads). A few minor bands were also detected in both non-AD and AD samples with all three sets of primers (arrows).

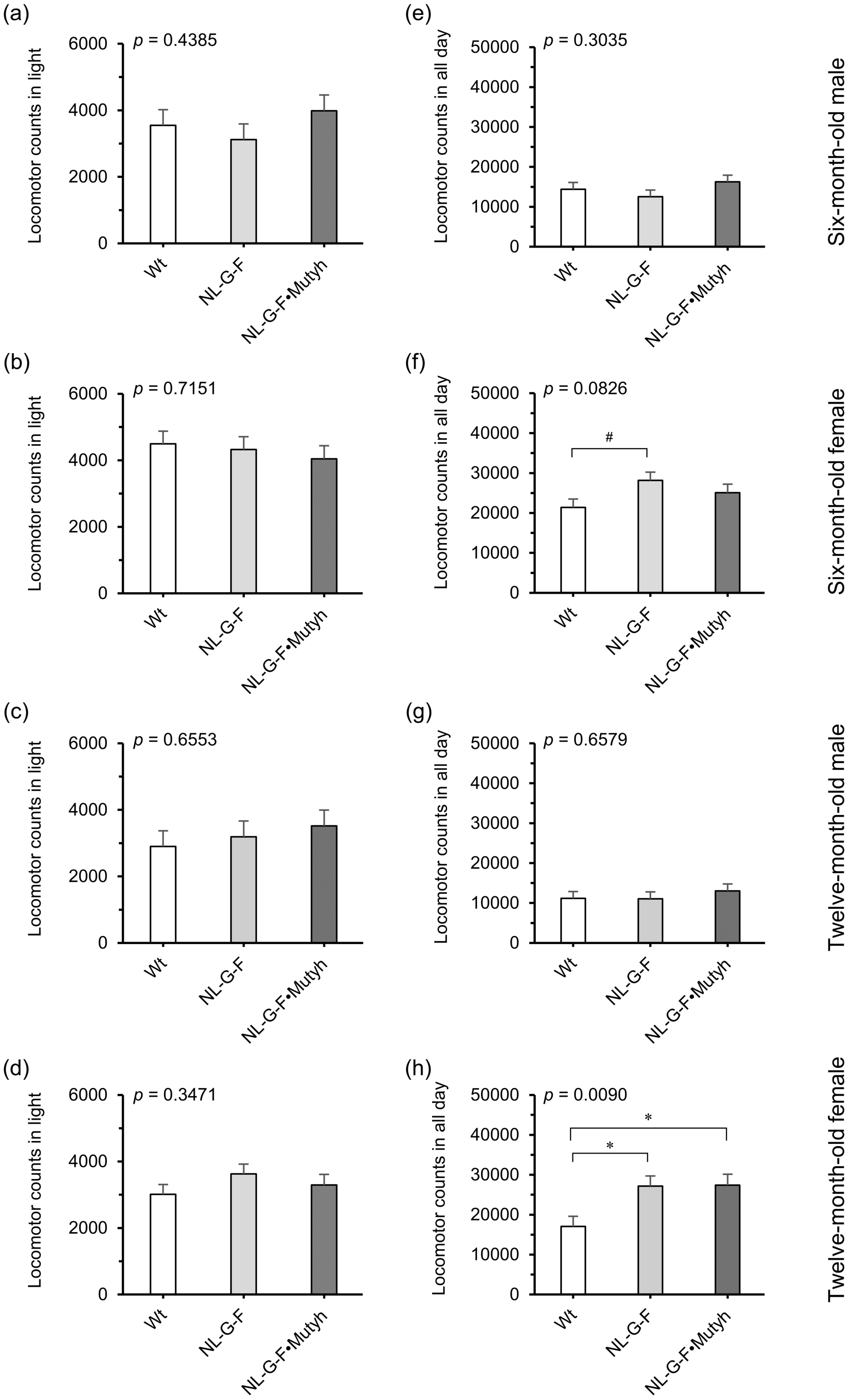
(a)
p = 0.4385
Locomotor counts in light
6000
4000
2000
0
Wt
NL-G-F
NL-G-F•Mutyh
(e)
p = 0.3035
Locomotor counts in all day
50000
40000
30000
20000
10000
0
Six-month-old male
(b)
p = 0.7151
(f)
p = 0.0826
#
Six-month-old female
(c)
p = 0.6553
(g)
p = 0.6579
Twelve-month-old male
(d)
p = 0.3471
(h)
p = 0.0090
*
*
Twelve-month-old female

Supplementary Fig. S3. Spontaneous locomotor activity of wild-type, *App*$^{NL-G-F/NL-G-F}$, *App*$^{NL-G-F/NL-G-F}$•*Mutyh*$^{-/-}$ mice. (a)-(d), Locomotor counts in the light phase (8:00 am to 8:00 pm, average counts over the three days) are shown. (e)-(h) Locomotor counts across the entire day are shown. Wild-type (Wt), *App*$^{NL-G-F/NL-G-F}$ (NL-G-F) and *App*$^{NL-G-F/NL-G-F}$•*Mutyh*$^{-/-}$ (NL-G-F•Mutyh). (a), (e) Six-month-old male mice. (b), (f) Six-month-old female mice. (c), (g) Twelve-month-old male mice. (d), (h) Twelve-month-old female mice. The data are expressed as the mean ± SEM, n = 13-15 per group. Statistical analyses were performed with a one-way ANOVA, and the *p* value is shown in each bar graph. In six-month-old female mice (f), a one-way ANOVA revealed marginal significance in all-day locomotor counts. Next, Hsu's multiple comparisons with the best (MCB) was performed. $^{\#}p < 0.05$. In twelve-month-old female mice (h), a one-way ANOVA revealed a significant difference in the all-day locomotor counts, and then a post-hoc Student's *t*-test was performed, $^{*}p < 0.05$.

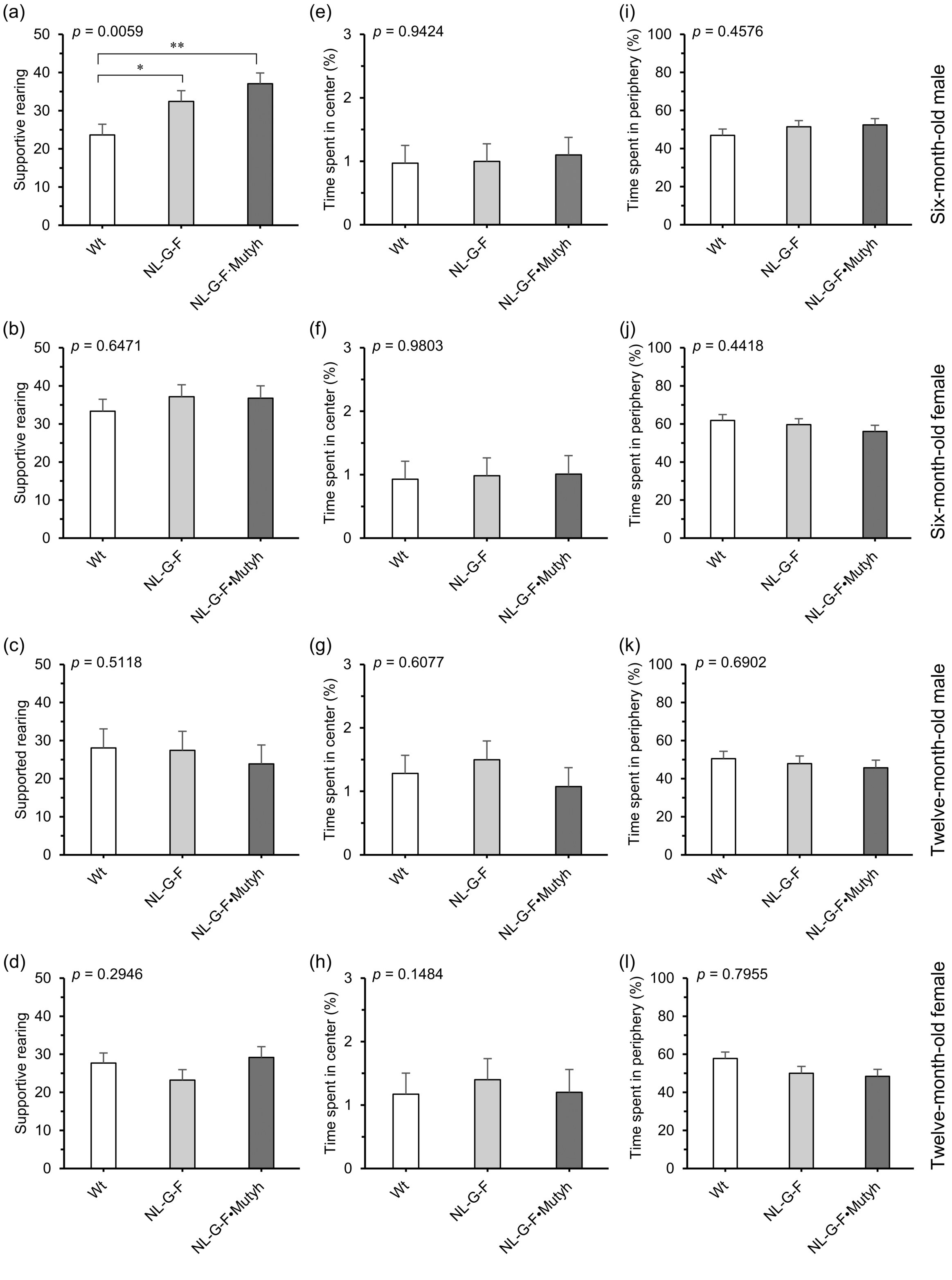
(a)
p = 0.0059
Supportive rearing
**
*
Wt
NL-G-F
NL-G-F·Mutyh
(b)
p = 0.6471
(c)
p = 0.5118
Supported rearing
(d)
p = 0.2946
(e)
p = 0.9424
Time spent in center (%)
(f)
p = 0.9803
(g)
p = 0.6077
(h)
p = 0.1484
(i)
p = 0.4576
Time spent in periphery (%)
(j)
p = 0.4418
(k)
p = 0.6902
(l)
p = 0.7955
NL-G-F•Mutyh
Six-month-old male
Six-month-old female
Twelve-month-old male
Twelve-month-old female

Supplementary Fig. S4. Open field test in wild-type, *App*$^{NL\text{-}G\text{-}F/NL\text{-}G\text{-}F}$ and *App*$^{NL\text{-}G\text{-}F/NL\text{-}G\text{-}F}$•*Mutyh*$^{-/-}$ mice. (a)-(d) Number with supportive rearing. (e)-(h) Time spent in the center zone. (i)-(l) Time spent in the periphery zone. Wild-type (Wt), *App*$^{NL\text{-}G\text{-}F/NL\text{-}G\text{-}F}$ (NL-G-F) and *App*$^{NL\text{-}G\text{-}F/NL\text{-}G\text{-}F}$•*Mutyh*$^{-/-}$ (NL-G-F•Mutyh). (a), (e), (i) Six-month-old male mice. (b), (f), (j) Six-month-old female mice. (c), (g), (k) Twelve-month-old male mice. (d), (h), (l) Twelve-month-old female mice. The data are expressed as the mean ± SEM, n = 13-15 per group. Statistical analyses were performed with a one-way ANOVA, and the *p* value is shown in each bar graph. In six-month-old male mice (a), a one-way ANOVA revealed a statistically significant difference in supporting rearing, and then a post-hoc Student's *t*-test was performed, $^{*}p < 0.05$, and $^{**}p < 0.005$.

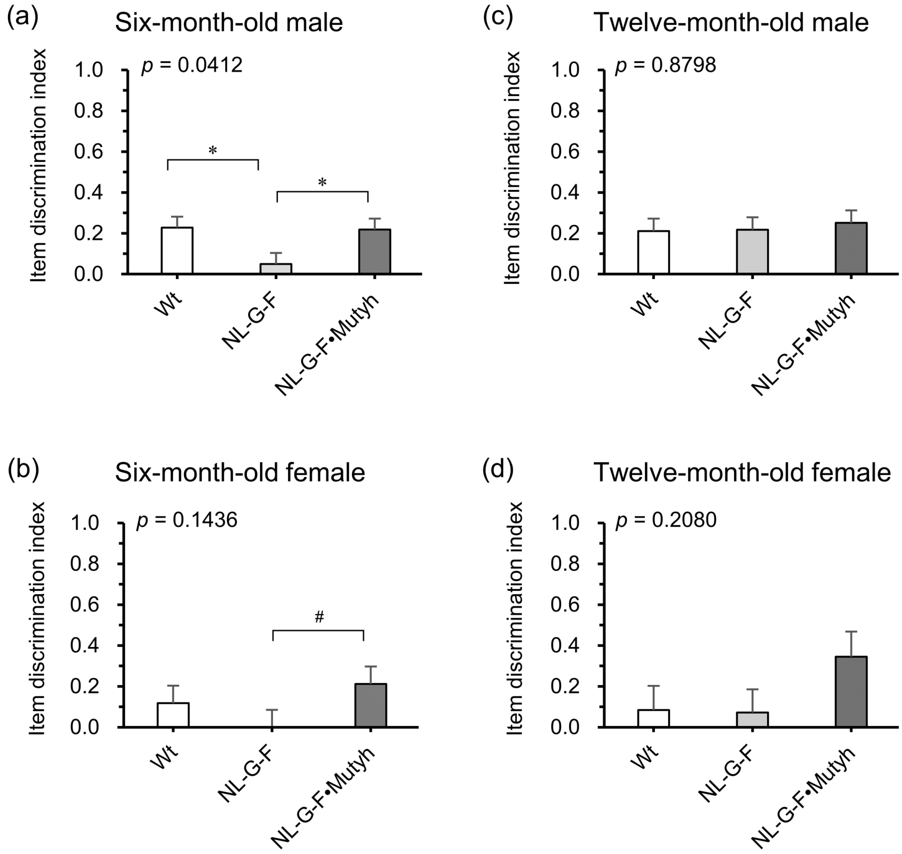

Supplementary Fig. S5. The item discrimination index during the novel object recognition test of wild-type, *App*$^{NL\text{-}G\text{-}F/NL\text{-}G\text{-}F}$, *App*$^{NL\text{-}G\text{-}F/NL\text{-}G\text{-}F}$•*Mutyh*$^{-/-}$ mice. (a) Six-month-old male mice. (b) Six-month-old female mice. (c) Twelve-month-old male mice. (d) Twelve-month-old female mice. Wild-type (Wt), *App*$^{NL\text{-}G\text{-}F/NL\text{-}G\text{-}F}$ (NL-G-F) and *App*$^{NL\text{-}G\text{-}F/NL\text{-}G\text{-}F}$•*Mutyh*$^{-/-}$ (NL-G-F•Mutyh). Item discrimination index = (periods contacting novel object – periods contacting familiar object) / (periods contacting novel object + periods contacting familiar object). The data are expressed as the mean ± SEM, n = 13-15 per group. Statistical analyses were performed with a one-way ANOVA, and the p value is shown in each bar graph. In six-month old male mice (a), a one-way ANOVA revealed a significant difference in the item discrimination index, and then a post-hoc Student's t-test was performed. $^{*}p < 0.05$. In six-month old female mice (b), a one-way ANOVA revealed marginal significant difference in the item discrimination index, and then Hsu's MCB was performed. $^{\#}p < 0.05$.

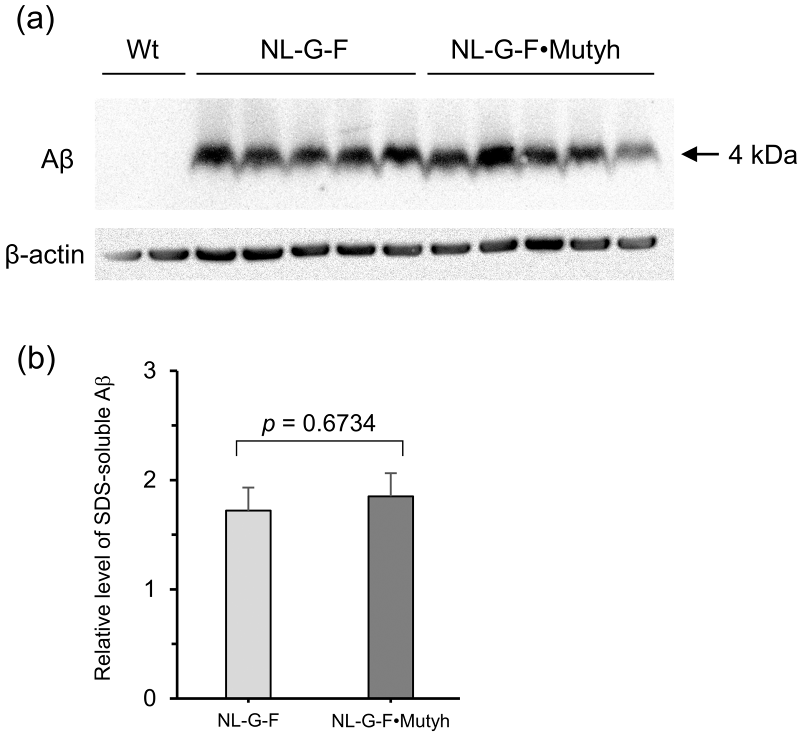

Supplementary Fig. S6. Western blot analyses of SDS-soluble Aβ peptide in six-month-old female mouse hippocampal extracts. (a) Representative Western blot with anti-Aβ (6E10). (b) A quantitative analysis of the Aβ levels in *App*$^{NL\text{-}G\text{-}F/NL\text{-}G\text{-}F}$ (NL-G-F), *App*$^{NL\text{-}G\text{-}F/NL\text{-}G\text{-}F}$•*Mutyh*$^{-/-}$ (NL-G-F•Mutyh) hippocampal extracts. The data were normalized by β-actin levels and expressed as the mean ± SEM, n = 5. Statistical analyses were performed with a one-way ANOVA, and the *p* value is shown in the bar graph. Similar levels of SDS-soluble Aβ peptide were detected in both hippocampal extracts.

(a)

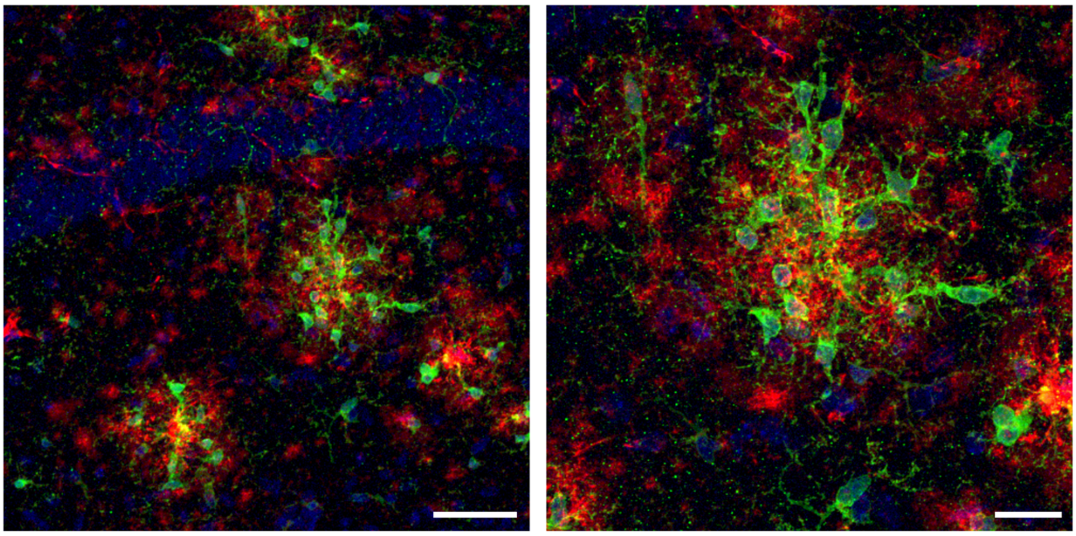

(b)

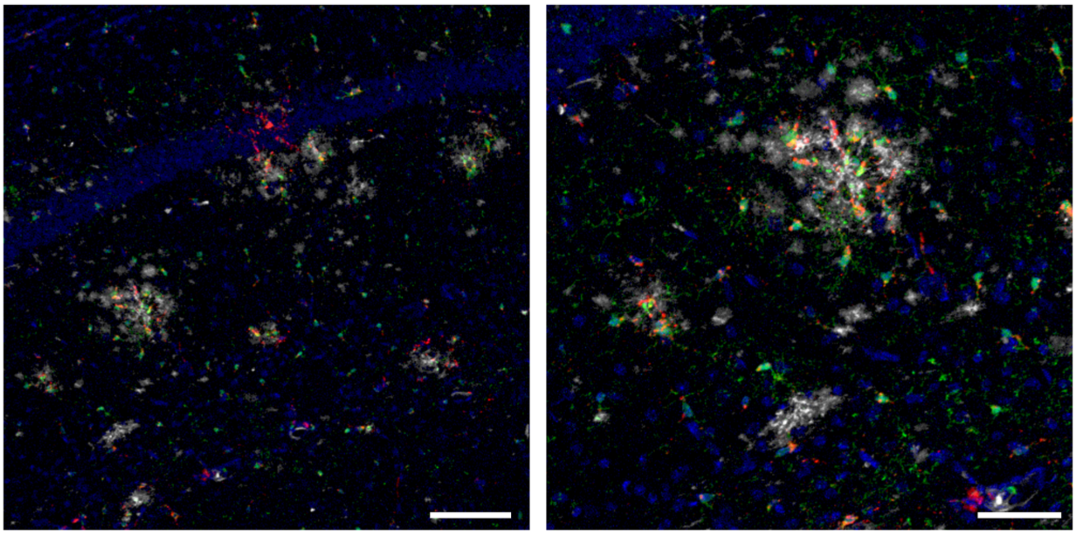

Supplementary Fig. S7. Microglia were highly clustered inside Aβ plaques in the *App*$^{\text{NL-G-F/NL-G-F}}$ hippocampus. (a) Double-immunofluorescence microscopy for Iba-1 (green) and Aβ (82E1, red) in hippocampus from six-month-old female *App*$^{\text{NL-G-F/NL-G-F}}$ mice. Nuclei were counter-stained by DAPI (blue). Scale bars = 50 μm (left) and 20 μm (right). (b) Triple-immunofluorescent microscopy for Iba-1 (green), CD68 (red), and Aβ (82E1, white) in hippocampus from six-month-old female *App*$^{\text{NL-G-F/NL-G-F}}$ mice. Nuclei were counter-stained by DAPI (blue). Scale bars = 100 μm (left) and 50 μm (right).
