# Supplementary material for: MUTYH Actively Contributes to Microglial Activation and Impaired Neurogenesis in the Pathogenesis of Alzheimer's Disease
Source: Oxid Med Cell Longev. 2021 Dec 21;2021:8635088. doi: 10.1155/2021/8635088 (PMC8714343; doi:10.1155/2021/8635088)
Supplement: Supplementary Materials — Figure S1: immunohistochemistry without a primary antibody as a negative control. Figure S2: multiforms of MUTYH mRNA detected in the human brain. Figure S3: spontaneous locomotor activity of wild-type, AppNL-G-F/NL-G-F, and AppNL-G-F/NL-G-F·Mutyh−/− mice. Figure S4: open-field test in wild-type, AppNL-G-F/NL-G-F, and AppNL-G-F/NL-G-F·Mutyh−/− mice. Figure S5: the item discrimination index during the novel object recognition test of wild-type, AppNL-G-F/NL-G-F, and AppNL-G-F/NL-G-F·Mutyh−/− mice. Figure S6: Western blot analyses of SDS-soluble Aβ peptide in six-month-old female mouse hippocampal extracts. Figure S7: immunofluorescence microscopy in the hippocampus from six-month-old female AppNL-G-F/NL-G-F mice. Table S1: list of human autopsy brain samples. Table S2: expression of multiforms of MUTYH mRNA in the human hippocampus with or without AD pathology. Table S3: the altered expression of marker genes for three types of astrocytes in the hippocampi of six-month-old female wild-type, AppNL-G-F/NL-G-F, and AppNL-G-F/NL-G-F·Mutyh−/− mice. Table S4: list of 103 genes subjected to functional annotation clustering by DAVID. [file 8635088.f1.zip › Mizuno_OMCL_Sup Table S1.pdf]

Supplementary Table S1: List of human autopsy brain samples.

| # | Diagnosis | Age (years) | Sex | CERAD score | Braak-NFT stage |
|---|---|---|---|---|---|
| 1 | AD | 87 | F | frequent | 6 |
| 2 | AD | 92 | F | frequent | 5 |
| 3 | AD | 91 | F | frequent | 5 |
| 4 | non-AD | 72 | F | sparse | 2 |
| 5 | non-AD | 72 | F | none | 3 |
| 6 | non-AD | 84 | F | none | 2 |

AD: Alzheimer’s disease.
