# Supplementary material for: MUTYH Actively Contributes to Microglial Activation and Impaired Neurogenesis in the Pathogenesis of Alzheimer's Disease
Source: Oxid Med Cell Longev. 2021 Dec 21;2021:8635088. doi: 10.1155/2021/8635088 (PMC8714343; doi:10.1155/2021/8635088)
Supplement: Supplementary Materials — Figure S1: immunohistochemistry without a primary antibody as a negative control. Figure S2: multiforms of MUTYH mRNA detected in the human brain. Figure S3: spontaneous locomotor activity of wild-type, AppNL-G-F/NL-G-F, and AppNL-G-F/NL-G-F·Mutyh−/− mice. Figure S4: open-field test in wild-type, AppNL-G-F/NL-G-F, and AppNL-G-F/NL-G-F·Mutyh−/− mice. Figure S5: the item discrimination index during the novel object recognition test of wild-type, AppNL-G-F/NL-G-F, and AppNL-G-F/NL-G-F·Mutyh−/− mice. Figure S6: Western blot analyses of SDS-soluble Aβ peptide in six-month-old female mouse hippocampal extracts. Figure S7: immunofluorescence microscopy in the hippocampus from six-month-old female AppNL-G-F/NL-G-F mice. Table S1: list of human autopsy brain samples. Table S2: expression of multiforms of MUTYH mRNA in the human hippocampus with or without AD pathology. Table S3: the altered expression of marker genes for three types of astrocytes in the hippocampi of six-month-old female wild-type, AppNL-G-F/NL-G-F, and AppNL-G-F/NL-G-F·Mutyh−/− mice. Table S4: list of 103 genes subjected to functional annotation clustering by DAVID. [file 8635088.f1.zip › Mizuno_OMCL_Sup Table S2.pdf]

Supplementary Table S2: Expression of multiforms of *MUTYH* mRNA in human hippocampus with or without AD pathology.

Transcript ID	Trascribed region[a]		1st exon	Splicing type in exon3	Additional features	RT-PCR product (base)[b]	MUTYH protein isoform No	Expression in AD hippocampus (TPM)[c]								Expression in non-AD hippocampus (TPM)[c]									
	start	end						1	2	3	4	5	6	7	8	9	10	11	12	13	14	15	16	17	18
NM_012222.3	45806189	45794914	α	1		413	1	0.105	0.000	0.138	0.153	0.224	0.000	0.777	0.000	0.000	0.000	0.000	0.000	0.000	0.000	0.000	0.206	0.000	0.010
NM_001293190.2	45805792	45794914	α	2		383	6	0.000	0.000	0.559	0.119	0.327	0.000	0.529	0.000	0.287	0.000	0.364	0.000	0.000	0.000	0.000	0.000	0.000	0.016
NM_001048171.2	45805792	45794914	α	3		380	2	0.100	0.235	0.944	0.278	0.565	0.000	0.373	0.000	0.257	0.000	0.503	0.000	1.866	0.000	0.000	0.576	0.000	0.000
NM_001293192.2	45805642	45794914	α	4		316	8	0.096	0.145	0.611	0.153	0.616	0.000	1.094	0.261	0.158	0.001	0.574	0.000	0.264	0.000	0.000	0.169	0.000	0.000
NM_001128425.2	45806189	45794914	α	5		422	5	0.173	0.236	0.227	0.274	0.127	0.000	0.126	0.000	0.077	0.150	0.829	0.076	0.000	0.036	0.286	0.076	0.000	0.017
NM_001293191.2	45806189	45794914	β	1		278	7	0.145	0.000	0.000	0.453	0.000	0.000	0.189	0.000	0.000	0.044	0.000	0.045	0.000	0.000	0.076	0.493	0.000	0.000
NM_001048174.2	45805642	45794914	β	3		245	4	2.176	0.193	0.000	0.391	0.000	0.000	0.122	0.000	0.000	0.007	0.621	0.000	0.000	3.582	0.000	1.207	1.377	0.000
NM_001293195.2	45806189	45794914	β	5	an extra exon after exon 1	395	4	0.113	0.000	0.319	0.199	0.044	0.000	0.108	0.000	0.052	0.256	0.183	0.210	0.065	0.163	0.201	0.103	0.031	0.000
NM_001048172.2	45805642	45794914	γ	2		316	3	0.000	0.000	0.000	0.251	0.071	0.000	0.438	0.000	0.248	0.000	0.000	0.293	0.000	0.000	0.000	0.000	1.492	0.010
NM_001048173.2	45805792	45794914	γ	3		313	4	0.000	0.230	0.000	1.039	0.516	0.000	0.354	0.000	0.244	0.000	0.000	0.423	0.000	0.816	0.000	1.244	1.572	0.007
NM_001293196.2	45806189	45794914	γ	4		249	8	0.000	0.128	0.000	0.540	0.742	0.000	0.332	0.000	0.136	0.000	0.000	0.176	0.000	0.132	0.000	0.266	0.218	0.000
NM_001350650.2	45806189	45794914	α	3	no exon 5	380	9	0.142	0.000	0.184	0.172	0.000	0.000	0.000	0.000	0.660	0.000	0.000	0.000	0.000	0.000	0.003	0.109	0.000	0.009
NM_001350651.2	45806189	45794914	α	4	no exon 5	316	9	0.104	0.000	0.171	0.136	0.000	0.000	0.000	0.000	0.155	0.000	0.000	0.000	0.000	0.000	0.000	0.100	0.000	0.000
NR_146882.2	45806189	45794914	α	5	an extra exon after exon 15	380	478 aa (52.7 kDa)	2.652	0.942	3.044	1.285	1.039	1.409	1.562	0.697	0.543	0.601	0.917	0.000	0.222	1.104	0.011	0.828	0.401	0.848
NR_146883.2	45805792	45794914	γ	3	an extra exon after exon 15	313	363 aa (39.6 kDa)	0.000	0.000	0.000	2.461	0.000	0.000	0.000	0.000	0.000	0.000	0.000	1.415	0.000	0.000	1.811	0.329	0.000	0.165
MSTRG.709.1	45806189	45794914	α	3	retained intron 5	380	349 aa (37.8 kDa)	0.103	1.401	1.635	1.145	0.585	0.296	0.248	0.000	1.677	0.655	0.521	0.000	0.000	0.172	0.000	0.407	0.000	0.000
MSTRG.709.2	45805792	45794914	γ	3	retained intron 5	313	349 aa (37.8 kDa)	0.000	1.182	0.000	2.015	0.644	0.000	0.244	0.000	0.496	3.638	0.000	0.314	0.000	1.886	0.000	0.851	1.496	0.010
MSTRG.709.3	45803976	45794914	novel 1st exon	4	retained introns 4, 5 and 8	NA[d]	267 aa (29.4 kDa)	2.491	0.988	1.799	0.910	0.711	1.130	1.044	0.429	0.915	0.719	3.025	1.770	0.129	1.115	1.346	1.114	0.333	0.201
MSTRG.709.4	45806189	45794914	α'	3	extended 1st exon (α'), retained introns 5 and 6	396	349 aa (37.8 kDa)	1.424	1.024	2.664	1.369	0.888	0.823	0.379	0.410	0.656	3.134	1.120	1.429	0.303	1.424	0.749	0.580	0.780	1.579
MSTRG.709.20	45806189	45794928	α	4	retained introns 4 and 11, shortened exon 16	316	NC[e]	0.128	0.184	0.621	0.861	1.105	0.373	1.512	0.380	0.439	0.625	0.820	0.317	0.094	0.000	3.508	0.284	0.000	0.648
MSTRG.709.21	45799266	45794929	exon 3	1	loss of exons 1 and 2, retained intron 11, shortened exon 16	NA[d]	NC[e]	2.270	1.335	0.536	4.552	0.597	0.000	0.241	1.499	0.511	0.000	0.340	2.397	0.443	1.058	0.000	0.661	0.706	0.000
Expression level of *MUTYH* at the gene level								19.014	9.871	20.975	24.481	11.123	5.608	12.592	5.251	10.345	18.471	13.686	13.208	4.207	15.086	15.368	12.140	9.776	4.551

[a]Transcribed region in human genome sequences [GRCh37/hg19].

[b]Sizes of RT-PCR products expected in Supplementary Fig. S2 are shown.

[c]Expression levels are shown by transcripts per million (TPM).

[d]NA = Not amplified.

[e]NC = non-coding.
