# Supplementary material for: MUTYH Actively Contributes to Microglial Activation and Impaired Neurogenesis in the Pathogenesis of Alzheimer's Disease
Source: Oxid Med Cell Longev. 2021 Dec 21;2021:8635088. doi: 10.1155/2021/8635088 (PMC8714343; doi:10.1155/2021/8635088)
Supplement: Supplementary Materials — Figure S1: immunohistochemistry without a primary antibody as a negative control. Figure S2: multiforms of MUTYH mRNA detected in the human brain. Figure S3: spontaneous locomotor activity of wild-type, AppNL-G-F/NL-G-F, and AppNL-G-F/NL-G-F·Mutyh−/− mice. Figure S4: open-field test in wild-type, AppNL-G-F/NL-G-F, and AppNL-G-F/NL-G-F·Mutyh−/− mice. Figure S5: the item discrimination index during the novel object recognition test of wild-type, AppNL-G-F/NL-G-F, and AppNL-G-F/NL-G-F·Mutyh−/− mice. Figure S6: Western blot analyses of SDS-soluble Aβ peptide in six-month-old female mouse hippocampal extracts. Figure S7: immunofluorescence microscopy in the hippocampus from six-month-old female AppNL-G-F/NL-G-F mice. Table S1: list of human autopsy brain samples. Table S2: expression of multiforms of MUTYH mRNA in the human hippocampus with or without AD pathology. Table S3: the altered expression of marker genes for three types of astrocytes in the hippocampi of six-month-old female wild-type, AppNL-G-F/NL-G-F, and AppNL-G-F/NL-G-F·Mutyh−/− mice. Table S4: list of 103 genes subjected to functional annotation clustering by DAVID. [file 8635088.f1.zip › Mizuno_OMCL_Sup Table S3.pdf]

Supplementary Table S3: The altered expression of marker genes for three types of astrocytes in the hippocampi of six-month-old female wild-type, *App* $^{NL-G-F/NL-G-F}$, *App* $^{NL-G-F/NL-G-F}$•*Mutyh* $^{-/-}$ mice.

Type of astrocyte	Marker gene	Relative expression (% wild-type)		*p* value[a]		
		App $^{NL-G-F/NL-G-F}$	*App* $^{NL-G-F/NL-G-F}$•*Mutyh* $^{-/-}$	*App* $^{NL-G-F/NL-G-F}$ vs. wild-type	*App* $^{NL-G-F/NL-G-F}$ vs. *App* $^{NL-G-F/NL-G-F}$•*Mutyh* $^{-/-}$	*App* $^{NL-G-F/NL-G-F}$ • *Mutyh* $^{-/-}$ vs. wild-type
PAN-reactive astrocytes	*Lcn2*	98.6	107.9	8.89E-01	1.90E-01	1.53E-01
	Steap4	100.0	90.8	9.41E-01	4.85E-01	4.42E-01
	S1pr3	94.6	95.3	2.89E-01	5.04E-01	6.77E-01
	Timp1	99.3	100.0	6.02E-01	6.12E-01	9.88E-01
	Hspb1	73.2	90.8	**1.86E-02**	5.07E-02	5.51E-01
	Cxcl10	114.1	144.4	7.16E-02	6.88E-01	**3.67E-02**
	Cd44	85.9	87.1	6.57E-01	4.19E-01	2.25E-01
	Osmr	128.3	107.9	**4.22E-02**	3.83E-01	1.80E-01
	Cp	96.6	95.3	7.50E-01	9.75E-01	7.27E-01
	Serpina3n	120.6	160.2	**1.16E-02**	**2.00E-04**	**7.52E-06**
	Aspg	105.7	96.6	5.37E-01	1.01E-01	2.65E-01
	Vim	102.8	100.7	4.21E-01	4.90E-01	9.04E-01
	Gfap	141.4	155.8	**3.00E-04**	1.94E-01	**6.55E-05**
	Mean	104.7	110.2	ns	ns	ns
	SD	17.2	24.6			
A1-specific astrocytes	*H2-T23*	111.0	119.8	5.06E-01	1.81E-01	6.13E-02
	Serping1	118.9	105.7	2.21E-01	3.67E-01	7.23E-01
	H2-D1	112.5	124.0	1.98E-01	6.84E-01	3.57E-01
	Ggta1	97.9	101.4	7.60E-01	5.07E-01	7.16E-01
	Iigp1	102.1	84.1	1.91E-01	3.40E-01	6.93E-01
	Gbp2	102.8	117.3	1.17E-01	3.27E-01	5.00E-01
	Fbln5	103.5	89.5	6.07E-01	3.81E-01	7.07E-01
	Ugt1a1	96.6	100.0	6.66E-01	8.26E-01	8.31E-01
	Fkbp5	92.0	102.8	1.17E-01	**4.82E-02**	5.93E-01
	Psmb8	124.0	118.9	6.76E-02	6.78E-01	**3.38E-02**
	Srgn	103.5	100.7	1.79E-01	9.11E-01	2.12E-01
	Amigo2	105.7	111.0	2.73E-01	6.27E-01	1.29E-01
	Mean	105.9	106.3	ns	ns	ns
	SD	8.9	11.8			
A2-specific astrocytes	*Clcf1*	100.7	100.7	8.38E-01	9.76E-01	8.62E-01
	Tgm1	100.0	100.7	5.99E-01	8.02E-01	7.81E-01
	Ptx3	103.5	105.7	9.43E-01	3.88E-01	4.26E-01
	S100a10	110.2	82.9	3.66E-01	1.31E-01	4.96E-01
	Sphk1	105.7	106.4	6.94E-01	8.93E-01	5.99E-01
	Cd109	100.0	100.0	6.42E-01	2.16E-01	1.04E-01
	Ptgs2	83.5	140.4	6.56E-01	**7.90E-03**	**1.63E-02**
	Emp1	101.4	109.4	2.32E-01	2.13E-01	9.54E-01
	Slc10a6	113.3	111.7	8.78E-02	8.50E-01	6.43E-02
	Tm4sf1	101.4	94.6	8.96E-01	4.87E-01	4.13E-01
	B3gnt5	103.5	104.3	7.74E-01	8.98E-01	6.79E-01
	Cd14	108.7	103.5	4.15E-01	8.15E-01	3.02E-01
	Mean	102.7	105.0	ns	ns	ns
	SD	7.1	12.9			
