# Supplementary material for: MUTYH Actively Contributes to Microglial Activation and Impaired Neurogenesis in the Pathogenesis of Alzheimer's Disease
Source: Oxid Med Cell Longev. 2021 Dec 21;2021:8635088. doi: 10.1155/2021/8635088 (PMC8714343; doi:10.1155/2021/8635088)
Supplement: Supplementary Materials — Figure S1: immunohistochemistry without a primary antibody as a negative control. Figure S2: multiforms of MUTYH mRNA detected in the human brain. Figure S3: spontaneous locomotor activity of wild-type, AppNL-G-F/NL-G-F, and AppNL-G-F/NL-G-F·Mutyh−/− mice. Figure S4: open-field test in wild-type, AppNL-G-F/NL-G-F, and AppNL-G-F/NL-G-F·Mutyh−/− mice. Figure S5: the item discrimination index during the novel object recognition test of wild-type, AppNL-G-F/NL-G-F, and AppNL-G-F/NL-G-F·Mutyh−/− mice. Figure S6: Western blot analyses of SDS-soluble Aβ peptide in six-month-old female mouse hippocampal extracts. Figure S7: immunofluorescence microscopy in the hippocampus from six-month-old female AppNL-G-F/NL-G-F mice. Table S1: list of human autopsy brain samples. Table S2: expression of multiforms of MUTYH mRNA in the human hippocampus with or without AD pathology. Table S3: the altered expression of marker genes for three types of astrocytes in the hippocampi of six-month-old female wild-type, AppNL-G-F/NL-G-F, and AppNL-G-F/NL-G-F·Mutyh−/− mice. Table S4: list of 103 genes subjected to functional annotation clustering by DAVID. [file 8635088.f1.zip › Mizuno_OMCL_Sup Table S4.pdf]

Supplementary Table S4: List of 103 genes subjected to functional annotation clustering by DAVID.

Transcript cluster ID	Gene symbol[a]	Average expressionlevels (log_2)			Standard Deviation of expression levels (log_2)			F-test	*App*$^{NL-G-F/NL-G-F}$ vs wild-type		*App*$^{NL-G-F/NL-G-F}$•*Mutyh*$^{-/-}$ vs *App*$^{NL-G-F/NL-G-F}$		*App*$^{NL-G-F/NL-G-F}$•*Mutyh*$^{-/-}$ vs wild-type	
		Wild-type	*App*$^{NL-G-F/NL-G-F}$	*App*$^{NL-G-F/NL-G-F}$ •*Mutyh*$^{-/-}$	Wild-type	*App*$^{NL-G-F/NL-G-F}$	*App*$^{NL-G-F/NL-G-F}$ •*Mutyh*$^{-/-}$	*p* value	Fold change	*p* value	Fold change	*p* value	Fold change	*p* value
17264835	*Cd68*	7.90	8.88	8.68	0.03	0.10	0.02	3.18E-07	1.97	1.21E-07	-1.15	4.80E-03	1.71	1.25E-06
17377464	*Cst7*	5.53	7.64	6.95	0.32	0.23	0.05	2.62E-06	4.33	9.18E-07	-1.61	8.90E-03	2.69	1.27E-05
17338416	*Trem2*	7.01	7.73	7.50	0.03	0.03	0.08	4.52E-06	1.65	1.73E-06	-1.17	2.46E-02	1.41	1.61E-05
17278328	*Serpina3n*	10.03	10.30	10.71	0.10	0.03	0.07	2.41E-05	1.20	1.16E-02	1.33	2.00E-04	1.60	7.52E-06
17359344	*Entpd1*	7.32	7.47	7.79	0.03	0.07	0.03	9.73E-05	1.11	3.08E-02	1.25	6.00E-04	1.39	3.12E-05
17498099	*Ctsd*	10.90	11.60	11.31	0.15	0.04	0.11	3.00E-04	1.63	8.71E-05	-1.23	3.40E-03	1.33	1.91E-02
17266960	*Ccl6*	4.78	5.90	5.46	0.36	0.10	0.16	3.00E-04	2.18	9.03E-05	-1.36	3.12E-02	1.61	2.30E-03
17475231	*Tmem145*	7.50	7.31	7.14	0.05	0.00	0.02	6.00E-04	-1.14	7.60E-03	-1.12	1.95E-02	-1.28	2.00E-04
17402433	*Arsj*	6.73	6.34	7.08	0.00	0.15	0.15	8.00E-04	-1.31	1.19E-02	1.67	2.00E-04	1.28	2.18E-02
17457971	*Olfr452*	5.69	5.40	5.14	0.12	0.10	0.12	8.00E-04	-1.22	3.81E-02	-1.20	6.40E-03	-1.47	2.00E-04
17439464	*Anxa3*	6.79	7.31	7.14	0.06	0.16	0.11	9.00E-04	1.43	3.00E-04	-1.12	2.25E-02	1.28	1.22E-02
17245223	*Lyz2*	5.96	7.10	6.67	0.21	0.37	0.08	1.30E-03	2.21	4.00E-04	-1.35	3.14E-02	1.64	1.42E-02
17217174	*Klhdc8a*	6.74	6.46	6.01	0.28	0.01	0.08	1.50E-03	-1.22	4.68E-02	-1.36	1.20E-02	-1.67	4.00E-04
17406186	*Tdo2*	5.77	5.56	5.99	0.01	0.07	0.12	1.60E-03	-1.16	2.36E-02	1.35	5.00E-04	1.17	2.48E-02
17250624	*Fam83g*	5.31	5.65	5.38	0.08	0.07	0.08	1.70E-03	1.26	8.00E-04	-1.21	2.50E-03	1.05	4.35E-01
17256215	*Wipf2*	8.71	8.43	8.65	0.04	0.04	0.06	1.80E-03	-1.21	6.00E-04	1.16	5.80E-03	-1.04	1.52E-01
17349884	*Pcdhb6*	7.83	7.68	8.01	0.04	0.04	0.06	1.80E-03	-1.11	4.14E-02	1.25	5.00E-04	1.13	1.70E-02
17479834	*Mex3b*	7.61	7.31	7.70	0.02	0.15	0.01	2.20E-03	-1.22	4.20E-03	1.31	9.00E-04	1.07	3.20E-01
17248809	*Havcr2*	5.79	6.11	6.33	0.10	0.14	0.22	2.20E-03	1.24	4.88E-02	1.17	1.91E-02	1.45	7.00E-04
17272509	*Mxra7*	7.05	7.37	7.16	0.06	0.10	0.04	2.20E-03	1.25	7.00E-04	-1.15	1.36E-02	1.08	7.16E-02
17384622	*Zbtb6*	7.60	7.38	7.61	0.03	0.06	0.06	2.80E-03	-1.17	4.60E-03	1.17	1.20E-03	1.01	3.77E-01
17248621	*Ccnjl*	7.19	7.06	7.45	0.14	0.01	0.04	3.00E-03	-1.09	2.54E-02	1.31	9.00E-04	1.20	5.39E-02
17361435	*Npas4*	7.94	7.37	6.86	0.40	0.17	0.13	3.20E-03	-1.48	4.83E-02	-1.42	3.09E-02	-2.11	1.00E-03
17490111	*Zfp715*	6.80	6.57	6.86	0.05	0.02	0.08	3.30E-03	-1.17	5.70E-03	1.22	1.40E-03	1.04	3.62E-01
17452311	*Cux2*	6.71	6.49	6.13	0.16	0.05	0.15	3.60E-03	-1.17	3.42E-02	-1.28	4.90E-02	-1.49	1.10E-03
17239638	*Pde7b*	9.07	8.92	9.25	0.09	0.05	0.10	3.70E-03	-1.11	1.57E-02	1.26	1.20E-03	1.14	1.22E-01
17262193	*Cdk2ap1*	7.42	7.14	6.99	0.11	0.11	0.12	4.20E-03	-1.21	4.34E-02	-1.11	4.65E-02	-1.34	1.20E-03
17298379	*Tnnc1*	6.43	6.71	6.29	0.06	0.18	0.09	4.30E-03	1.22	4.06E-02	-1.33	1.30E-03	-1.10	5.12E-02
17233629	*Psap*	11.69	11.88	11.63	0.03	0.01	0.02	4.60E-03	1.13	7.60E-03	-1.18	2.00E-03	-1.04	3.86E-01
17371520	*Mettl5os*	5.99	5.70	6.13	0.00	0.07	0.17	4.70E-03	-1.23	1.82E-02	1.34	1.50E-03	1.10	1.37E-01
17332956	*Rnaset2b*	9.71	10.30	9.80	0.18	0.09	0.19	4.80E-03	1.50	4.10E-03	-1.41	3.00E-03	1.06	8.40E-01
17340720	*Rnaset2a*	9.71	10.28	9.80	0.18	0.07	0.19	5.30E-03	1.48	4.70E-03	-1.39	3.20E-03	1.06	8.02E-01
17280054	*Trib2*	7.18	7.04	7.29	0.02	0.04	0.02	5.60E-03	-1.10	3.35E-02	1.19	1.70E-03	1.08	8.80E-02
17356685	*Gpha2*	5.81	6.04	5.73	0.11	0.05	0.09	5.90E-03	1.17	5.40E-03	-1.24	3.50E-03	-1.06	7.74E-01
17374047	*Kcna4*	8.24	8.04	8.31	0.03	0.04	0.08	6.30E-03	-1.15	1.70E-02	1.21	2.20E-03	1.05	2.16E-01
17325109	*Itgb5*	8.61	8.95	8.81	0.06	0.04	0.12	6.40E-03	1.27	2.00E-03	-1.11	3.06E-02	1.14	1.13E-01
17530733	*Grm2*	9.88	9.50	10.08	0.03	0.07	0.21	6.90E-03	-1.30	1.28E-02	1.50	2.70E-03	1.15	3.36E-01
17389066	*Dnajc24*	6.54	6.32	6.50	0.03	0.03	0.04	7.40E-03	-1.16	4.60E-03	1.14	6.10E-03	-1.03	8.58E-01
17218694	*Ankrd45*	9.04	8.77	9.01	0.08	0.07	0.07	9.50E-03	-1.21	5.10E-03	1.18	9.20E-03	-1.03	7.15E-01
17376626	*Mcm8*	5.91	5.69	5.90	0.12	0.02	0.05	1.04E-02	-1.17	4.10E-03	1.16	1.85E-02	-1.01	3.59E-01
17514789	*Gpr83*	7.51	7.36	7.58	0.11	0.11	0.06	1.19E-02	-1.11	1.38E-02	1.16	5.60E-03	1.05	5.80E-01
17315902	*Spef2*	5.94	6.33	5.92	0.18	0.11	0.10	1.19E-02	1.30	9.30E-03	-1.32	7.50E-03	-1.01	8.99E-01
17466624	*Tmem176b*	8.03	8.48	8.27	0.19	0.07	0.09	1.21E-02	1.37	4.00E-03	-1.16	3.96E-02	1.18	1.82E-01
17495821	*Cdr2*	6.95	7.40	7.10	0.24	0.11	0.15	1.29E-02	1.37	5.30E-03	-1.24	1.96E-02	1.11	4.30E-01
17314888	*Gpd1*	8.98	8.70	9.09	0.11	0.11	0.08	1.31E-02	-1.22	3.63E-02	1.31	4.50E-03	1.07	2.20E-01
17503841	*Lpcat2*	7.48	7.71	7.57	0.07	0.05	0.05	1.46E-02	1.17	5.70E-03	-1.10	2.49E-02	1.06	3.77E-01
17239142	*Samd5*	8.21	7.97	8.10	0.06	0.07	0.04	1.47E-02	-1.18	5.10E-03	1.09	3.75E-02	-1.08	2.42E-01
17282563	*Npc2*	8.43	8.66	8.43	0.04	0.12	0.13	1.47E-02	1.18	5.70E-03	-1.17	2.54E-02	1.00	3.71E-01
17346599	*Rpl7a-ps5*	7.56	7.89	7.61	0.16	0.10	0.16	1.74E-02	1.25	7.60E-03	-1.21	2.27E-02	1.04	5.06E-01
17431272	*Pdik1l*	6.96	6.79	6.99	0.08	0.03	0.10	1.75E-02	-1.13	1.45E-02	1.15	1.00E-02	1.02	8.21E-01
17478263	*Tmem86a*	7.74	7.98	7.64	0.12	0.09	0.07	1.76E-02	1.18	3.43E-02	-1.26	6.60E-03	-1.07	3.27E-01
17509139	*Ankrd37*	6.12	5.84	6.20	0.05	0.11	0.12	1.80E-02	-1.21	1.92E-02	1.28	8.70E-03	1.06	6.32E-01
17306793	*Tinf2*	6.29	5.99	6.20	0.13	0.03	0.08	1.83E-02	-1.23	7.30E-03	1.16	2.86E-02	-1.06	4.14E-01
17503118	*Mir27a*	5.72	5.99	5.81	0.13	0.05	0.03	1.88E-02	1.20	6.60E-03	-1.13	4.54E-02	1.06	2.58E-01
17318100	*Ly6c2*	5.72	5.92	5.72	0.09	0.09	0.02	1.90E-02	1.15	7.80E-03	-1.14	2.79E-02	1.00	4.45E-01
17349868	*Pcdhb2*	6.33	6.09	6.65	0.13	0.19	0.18	2.04E-02	-1.18	4.17E-02	1.47	7.40E-03	1.25	3.09E-01
17502378	*Tpm4*	7.75	7.40	7.75	0.10	0.13	0.10	2.14E-02	-1.28	1.35E-02	1.28	1.57E-02	1.00	9.25E-01
17257100	*Fmnl1*	8.23	8.12	8.33	0.06	0.10	0.10	2.18E-02	-1.08	2.75E-02	1.15	9.50E-03	1.06	5.24E-01
17350909	*Chsy3*	7.26	7.02	7.19	0.02	0.06	0.03	2.20E-02	-1.18	8.90E-03	1.12	3.31E-02	-1.05	4.32E-01
17541681	*Gpc3*	5.63	5.76	5.52	0.02	0.08	0.08	2.22E-02	1.10	4.63E-02	-1.18	8.10E-03	-1.08	3.04E-01
17320337	*Plxnb2*	8.05	8.37	8.03	0.14	0.05	0.16	2.36E-02	1.24	4.26E-02	-1.26	8.90E-03	-1.02	3.56E-01
17366886	*Mir467e*	7.62	8.01	7.75	0.26	0.12	0.13	2.36E-02	1.31	1.01E-02	-1.20	3.06E-02	1.09	5.06E-01
17510351	*Tmem221*	5.67	5.93	5.76	0.06	0.06	0.12	2.44E-02	1.20	2.03E-02	-1.13	1.36E-02	1.06	8.06E-01
17235837	*Zfp873*	6.14	5.78	5.94	0.11	0.16	0.29	2.47E-02	-1.28	1.49E-02	1.12	1.86E-02	-1.15	8.92E-01
17509891	*Ndufa13*	7.01	6.82	6.96	0.04	0.04	0.07	2.49E-02	-1.15	9.10E-03	1.11	4.96E-02	-1.04	3.19E-01
17447610	*Msx1*	6.58	6.97	6.82	0.13	0.08	0.15	2.51E-02	1.31	9.90E-03	-1.12	3.97E-02	1.18	4.09E-01
17511069	*Nanos3*	5.67	5.43	5.71	0.13	0.05	0.13	2.57E-02	-1.19	4.26E-02	1.22	1.00E-02	1.03	3.90E-01
17315245	*Krt18*	6.24	6.63	6.27	0.39	0.15	0.22	2.65E-02	1.31	1.16E-02	-1.28	3.24E-02	1.02	5.40E-01
17398970	*Mef2d*	10.45	10.31	10.60	0.06	0.02	0.11	2.66E-02	-1.10	3.82E-02	1.22	1.08E-02	1.11	4.53E-01
17250178	*Hist3h2a*	7.50	7.24	7.56	0.24	0.07	0.06	2.80E-02	-1.20	3.99E-02	1.25	1.14E-02	1.04	4.58E-01
17351811	*Acaa2*	6.95	7.34	6.95	0.14	0.06	0.26	2.80E-02	1.31	1.37E-02	-1.31	2.76E-02	1.00	6.75E-01
17466687	*Osbpl3*	7.08	6.77	7.11	0.04	0.14	0.12	2.91E-02	-1.24	3.39E-02	1.26	1.29E-02	1.02	5.64E-01
17276906	*Smoc1*	6.74	6.83	6.66	0.05	0.14	0.01	2.92E-02	1.06	3.40E-02	-1.13	1.30E-02	-1.06	5.66E-01
17260786	*Etaa1*	6.78	6.56	6.76	0.08	0.07	0.06	3.02E-02	-1.16	2.57E-02	1.15	1.63E-02	-1.01	7.83E-01
17281485	*Rps29*	5.85	6.11	5.83	0.12	0.19	0.02	3.03E-02	1.20	3.59E-02	-1.22	1.34E-02	-1.01	5.55E-01
17294002	*Zfp748*	6.64	6.44	6.62	0.04	0.13	0.06	3.05E-02	-1.15	2.21E-02	1.13	1.87E-02	-1.02	9.20E-01
17212904	*Mars2*	7.03	6.83	7.02	0.03	0.06	0.02	3.08E-02	-1.15	1.38E-02	1.15	3.54E-02	-1.00	5.74E-01
17248304	*Ubtd2*	7.71	7.59	7.79	0.13	0.08	0.07	3.08E-02	-1.08	2.28E-02	1.14	1.85E-02	1.05	8.99E-01
17464672	*Slc25a13*	6.16	6.35	5.98	0.19	0.12	0.14	3.13E-02	1.14	3.29E-02	-1.29	1.47E-02	-1.13	6.29E-01
17441524	*Hrk*	7.28	7.08	7.41	0.08	0.13	0.08	3.23E-02	-1.15	3.19E-02	1.26	1.57E-02	1.09	6.70E-01
17351878	*Zbtb7c*	5.62	5.80	5.58	0.09	0.09	0.14	3.25E-02	1.13	3.76E-02	-1.17	1.44E-02	-1.03	5.67E-01
17544196	*Hdx*	6.84	6.62	6.86	0.09	0.11	0.07	3.36E-02	-1.16	1.98E-02	1.18	2.52E-02	1.02	8.86E-01
17265646	*Tekt1*	5.99	6.22	5.96	0.07	0.08	0.11	3.37E-02	1.17	3.57E-02	-1.20	1.56E-02	-1.03	6.21E-01
17273116	*Oxld1*	5.60	5.77	5.64	0.04	0.05	0.01	3.40E-02	1.12	1.55E-02	-1.10	3.74E-02	1.03	5.98E-01
17299341	*Fbxo34*	8.01	7.87	8.03	0.02	0.04	0.04	3.43E-02	-1.10	4.11E-02	1.12	1.49E-02	1.02	5.46E-01
17363559	*Fam189a2*	6.94	7.12	6.79	0.23	0.05	0.21	3.60E-02	1.14	2.78E-02	-1.26	2.06E-02	-1.11	8.55E-01
17519357	*Onecut1*	6.04	5.74	6.10	0.10	0.17	0.12	3.63E-02	-1.23	3.10E-02	1.28	1.92E-02	1.04	7.73E-01
17262783	*Slc22a4*	6.33	6.54	6.30	0.15	0.04	0.09	3.77E-02	1.16	4.31E-02	-1.18	1.67E-02	-1.02	5.73E-01
17498897	*Tnfsf13b*	5.79	5.98	5.83	0.14	0.11	0.05	3.93E-02	1.14	1.69E-02	-1.10	4.76E-02	1.03	5.38E-01
17416967	*Spata6*	6.20	6.54	6.28	0.15	0.17	0.13	3.98E-02	1.26	1.89E-02	-1.20	3.97E-02	1.05	6.57E-01
17274940	*Atxn7l1*	9.31	9.13	9.30	0.09	0.08	0.05	4.00E-02	-1.13	2.84E-02	1.13	2.42E-02	-1.00	9.24E-01
17484925	*Cracr2b*	5.52	5.68	5.55	0.08	0.06	0.05	4.01E-02	1.12	1.81E-02	-1.09	4.36E-02	1.03	6.01E-01
17476199	*Zfp790*	7.78	7.65	7.84	0.06	0.05	0.05	4.04E-02	-1.09	3.51E-02	1.14	2.09E-02	1.04	7.56E-01
17292598	*Diras2*	10.05	9.74	10.12	0.06	0.18	0.11	4.24E-02	-1.23	4.90E-02	1.30	1.86E-02	1.05	5.64E-01
17290074	*Rpl34-ps2*	9.82	9.97	9.80	0.09	0.04	0.04	4.25E-02	1.11	3.22E-02	-1.13	2.43E-02	-1.02	8.65E-01
17391287	*Prom2*	5.49	5.69	5.42	0.05	0.12	0.11	4.30E-02	1.14	3.95E-02	-1.20	2.14E-02	-1.05	7.13E-01
17407138	*Il6ra*	7.12	7.29	7.03	0.13	0.06	0.10	4.47E-02	1.13	3.50E-02	-1.20	2.47E-02	-1.07	8.36E-01
17318895	*Foxred2*	7.11	6.92	7.07	0.03	0.05	0.05	4.67E-02	-1.14	2.83E-02	1.11	3.26E-02	-1.03	9.33E-01
17465711	*Akr1b3*	6.26	5.85	6.25	0.14	0.30	0.20	4.78E-02	-1.33	2.52E-02	1.32	3.97E-02	-1.00	7.85E-01
17274670	*Myt1l*	9.73	9.52	9.70	0.08	0.05	0.03	4.89E-02	-1.15	3.51E-02	1.13	2.88E-02	-1.02	9.07E-01
17233699	*Rpl27a-ps1*	6.11	6.37	6.13	0.13	0.11	0.15	4.89E-02	1.20	3.02E-02	-1.18	3.34E-02	1.02	9.53E-01
17523126	*Ttc21a*	6.04	6.42	5.98	0.14	0.11	0.27	4.89E-02	1.30	4.74E-02	-1.36	2.33E-02	-1.04	6.71E-01
17527565	*Csk*	6.77	6.88	6.73	0.10	0.06	0.02	4.93E-02	1.08	2.33E-02	-1.11	4.82E-02	-1.03	6.65E-01

[a]Among all transcript clusters (34,472), 1,164 with a raw expression intensity of >5.64 in log_2 exhibited significantly differing expression among wild-type, *App*$^{NL-G-F/NL-G-F}$ and *App*$^{NL-G-F/NL-G-F}$•*Mutyh*$^{-/-}$ hippocampus (ANOVA with eBayes correction, F-test $p < 0.05$). Among them, 138 clusters exhibited significantly different expression between wild-type vs. *App*$^{NL-G-F/NL-G-F}$ and *App*$^{NL-G-F/NL-G-F}$ vs. *App*$^{NL-G-F/NL-G-F}$•*Mutyh*$^{-/-}$ samples. Among those 138 clusters, 103 genes were functionally annotated with an offcial gene symbol.
